# Integrated multi-omic analysis characterizes an IFNγ-responsive-to-myeloid-suppressive immune continuum in immune-active hepatocellular carcinoma

**DOI:** 10.1371/journal.pone.0359327

**Published:** 2026-10-06

**Authors:** Luping Lin, Ningna Weng, Huaying Lai, Guangfeng Wu, Jinfeng Lin, Xinjian Wu, Sha Huang, Xiao Chen

**Affiliations:** 1 Department of Hepatobiliary and Pancreatic Oncology, Clinical Oncology School of Fujian Medical University, Fujian Cancer Hospital, Fuzhou, China; 2 Clinical Oncology School of Fujian Medical University, Fujian Cancer Hospital, Fuzhou, China; Huazhong Agriculture University, CHINA

## Abstract

Immune-active hepatocellular carcinoma (HCC) is commonly treated as a single inflamed phenotype, although its internal immune heterogeneity remains incompletely defined. We integrated public bulk transcriptomic, single-cell, spatial transcriptomic, proteomic, phosphoproteomic, and treatment-cohort data to test whether this heterogeneity is better represented by discrete states or a continuous immune axis. In 112 checkpoint-high TCGA-LIHC tumors, an inflamed-minus-myeloid axis showed structured heterogeneity but no robust empirical bimodality: the observed kernel-density curve had one prominent mode and none of 1,000 bootstrap resamples contained two or more prominent modes. IFNγ-responsive-associated and myeloid-suppressive-associated labels were therefore retained only as operational summaries of opposing axis tendencies, and the biological polarity persisted across alternative definitions of immune-active entry. A fixed 10-gene within-sample rank template-contrast score transferred the axis to independent HCC cohorts. Transcriptomic analyses based on gene sets non-overlapping with the compact panel and original state-defining genes supported the IFNγ-oriented end, whereas spatial analyses showed local cross-end coexistence with reduced immediate-neighbor mixing relative to a count-preserving random-label null, and paired proteomic/phosphoproteomic analyses showed concordant myeloid-oriented associations involving macrophage, fibroblast/extracellular-matrix, hypoxia, SPP1-integrin-FAK, and myeloid-receptor programs. Direct application of the frozen transfer score to four public HCC immunotherapy cohorts yielded heterogeneous, imprecise response associations (pooled Hedges’ g = 0.10, 95% CI −0.42 to 0.61) and did not establish clinical response prediction. These results support a structured IFNγ-responsive-to-myeloid-suppressive continuum within immune-active HCC. The framework is transferable across public datasets but requires prospective HCC-specific clinical validation, while functional perturbation studies are needed before causal or therapeutic claims.

## Introduction

Hepatocellular carcinoma (HCC) remains a major cause of cancer-related mortality, and most patients with intermediate or advanced disease rely on systemic therapy. Although immune checkpoint inhibitors (ICIs) and ICI-based combinations have expanded treatment options for HCC, objective response rates remain limited and clinically robust biomarkers for treatment selection are still lacking [1–3]. This challenge is particularly relevant in HCC, where tumors arise in the setting of chronic inflammation and cirrhosis and are shaped by interacting tumor, lymphoid, myeloid, stromal, endothelial, and hepatocyte-associated programs [4].

Previous studies provided a biological basis for considering opposing immune tendencies within immune-active HCC, but did not establish that these tendencies form two naturally discrete patient populations. Sia et al. identified an immune-specific HCC class that could itself be subdivided into an adaptive T-cell-response subtype and an exhausted subtype enriched for TGFβ1-regulated immunosuppressive programs [5]. More recently, single-cell profiling of liver cancer by Xue et al. resolved distinct tumor immune microenvironment ecologies, including immune activation and myeloid- or stromal-mediated immune suppression [6], while other single-cell studies further demonstrated extensive lymphoid, myeloid, stromal, and malignant-cell heterogeneity within the HCC ecosystem [7]. Together, these observations suggested that immune-active tumors may differ not simply in the overall amount of immune infiltration, but in the balance between IFNγ-linked lymphoid effector competence and countervailing myeloid/stromal suppression. This literature therefore motivated a two-pole working hypothesis while leaving the underlying population structure unresolved.

Accordingly, we tested whether internal heterogeneity within immune-active HCC could be organized along an IFNγ-responsive-to-myeloid-suppressive immune axis without prespecifying that the two ends constituted naturally discrete populations. The analytical workflow comprised five linked stages: (i) operational identification of immune-active HCC, continuity/modality assessment, entry-definition sensitivity analyses, and strict non-overlap bulk characterization; (ii) derivation and frozen external transfer of a compact rank-based template-contrast rule that yields a continuous signed transfer score and optional axis-associated labels; (iii) complementary sample-aware single-cell, spatial, proteomic, and phosphoproteomic characterization, with transcriptomic support from gene sets non-overlapping with the compact panel and original state-defining genes distinguished from analyses that reuse state-defining genes, and with cross-modal evidence evaluated separately; (iv) treatment-relevance analysis comprising a GSE206325 responder-program bridge and direct frozen evaluation in four public HCC immunotherapy cohorts, with cross-cancer cohorts retained only as secondary context; and (v) upstream multi-omic anchoring in TCGA-LIHC. The study was designed to characterize the reproducibility and cross-dataset transferability of a literature-motivated biological continuum, not to establish a ground-truth categorical taxonomy, causal mechanism, therapeutic target, or clinically validated response predictor. An overview of the analytical framework is shown in Fig 1.

**Fig 1:**
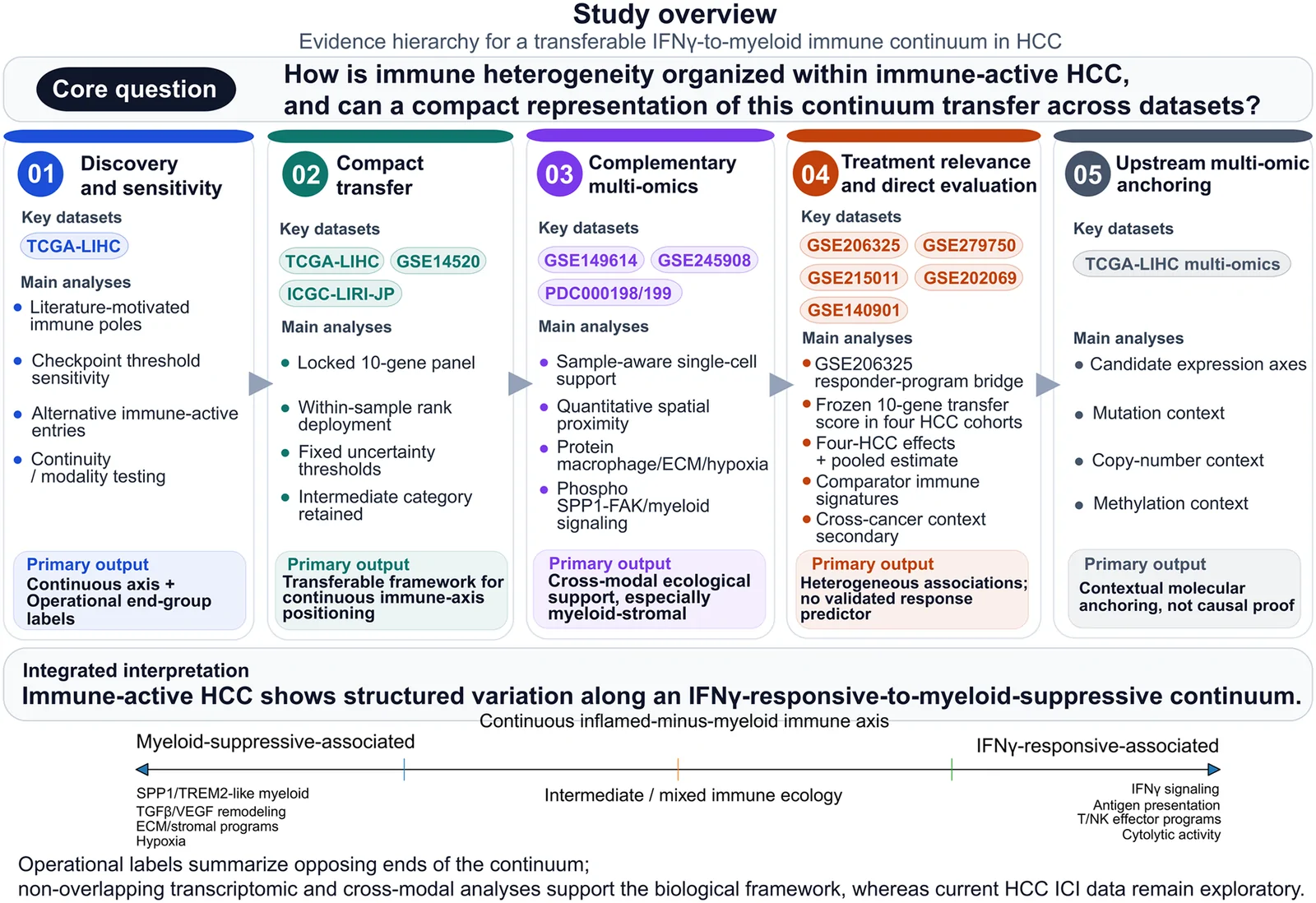
Study overview of the transferable multi-omic framework for an IFNγ-responsive-to-myeloid-suppressive immune continuum in HCC. The schematic summarizes the continuous inflamed-minus-myeloid axis and five analytical stages: TCGA-LIHC discovery, continuity/modality assessment, entry-definition sensitivity analyses, and strict non-overlap bulk characterization; frozen compact-panel transfer to external HCC cohorts; complementary sample-aware single-cell, spatial, proteomic, and phosphoproteomic characterization; treatment-relevance mapping using a GSE206325 responder-program bridge together with direct frozen evaluation in four HCC immunotherapy cohorts; and upstream TCGA multi-omic anchoring. IFNγ-responsive-associated and myeloid-suppressive-associated labels denote operational tendencies toward opposing ends of the continuum, with intermediate or mixed ecologies retained. Treatment-cohort findings are exploratory and are not presented as a validated clinical response predictor.

## Materials and methods

### Public datasets and overall study design

This study characterized immune heterogeneity within immune-active hepatocellular carcinoma (HCC) using exclusively public, de-identified data. Discovery and continuity analyses were performed in TCGA-LIHC [8], while GSE14520 [9] and ICGC-LIRI-JP [10] were used for frozen external HCC transfer. Complementary cellular and cross-modal characterization used GSE149614 [7] for sample-aware single-cell analysis, GSE245908 [11] for spatial localization and proximity analysis, and the paired PDC000198/PDC000199 datasets [12] for proteomic and phosphoproteomic analysis. GSE206325 [13] was used only to derive treatment-associated responder programs. Direct frozen treatment evaluation used GSE140901 [14], GSE215011 [15], GSE279750 [16], and GSE202069 [17]. GSE285963 [18] was used only for a supportive paired pre-/later-treatment analysis, whereas GSE78220 [19], GSE91061 [20], IMvigor210 [21], and TCGA-CESC [22] were retained only as secondary cross-cancer context and were not used to tune or validate the frozen HCC transfer rule. Throughout the manuscript, ‘IFNγ-responsive-associated’ and ‘myeloid-suppressive-associated’ denote operational patient-level labels derived from opposing axis tendencies. ‘IFNγ-like’ and ‘myeloid-like’ denote projected directional labels in single-cell analyses, whereas ‘IFNγ-high’ and ‘myeloid-high’ denote spatial spot-level extremes; proteomic and phosphoproteomic analyses are described by orientation along the corresponding partial molecular axes. To our knowledge, no externally validated reference classification exists for this specific IFNγ-to-myeloid HCC immune continuum. Accordingly, the axis was treated as a biological construct rather than as a ground-truth clinical class. Support for this biological construct was evaluated through prespecified biological direction, sensitivity to alternative immune-active definitions, frozen transfer to independent HCC cohorts, strict non-overlap transcriptomic analyses, complementary single-cell and spatial localization analyses, and cross-modal proteomic and phosphoproteomic evidence. None of these analyses was interpreted as proof of causality or as a substitute for prospective functional validation. The two Visium tumor sections reanalyzed from GSE245908 were GSM7850822 (CHC20) and GSM7850823 (CHC23), which constitute the spatial-transcriptomics subseries used in this study [11]. The source investigators reported that HCC and adjacent non-tumoral tissues were obtained from patients undergoing liver resection at Haut-Lévêque Hospital, Pessac, France; patients were informed about the secondary research use of their data and tissue samples and gave consent; and the source study was approved by the Health and Research Ethics Center of Bordeaux (reference CER-BDX 2024−10) [11]. The present study was a secondary analysis of publicly available, de-identified datasets and involved no new participant recruitment or sample collection. The present authors did not access identifiable private information. Accordingly, no additional institutional ethics approval or informed consent was required for the present secondary analysis.

Dataset and analysis provenance were recorded explicitly for this study. For each dataset, we documented the public accession and source study, data modality, biological or analytical sample unit, analyzed subset, endpoint or paired contrast, normalization and standardization scope, assigned analytical role, and the principal preprocessing or scoring rule used for the reported result. These records are summarized in Supplementary Table S1 in S2 File, section S1E, and in the machine-readable provenance_manifest.tsv supplied with the analysis files. Where a dataset contributed to more than one analysis, the roles and relevant subsets are distinguished. No external treatment cohort was used to reselect genes, change score direction, optimize thresholds, redefine response, or otherwise retune the frozen transfer rule.

### TCGA-LIHC preprocessing and endpoint harmonization

Expression matrices, annotations, and clinical data were obtained from a harmonized local TCGA-LIHC repository. One primary tumor per patient was retained. Because progression-free interval (PFI) could not be reconstructed reliably from the indexed local table alone, endpoints were harmonized with TCGA-CDR using submitter barcodes. PFI/PFI.time were used for recurrence-oriented analyses and overall survival (OS) was retained as supportive context [23].

### Immune-axis definition, operational labels, and sensitivity analyses

Prespecified checkpoint, inflamed, myeloid, antigen-presentation, and T-cell-support modules were scored from curated gene sets. The inflamed and myeloid modules were prespecified before any survival or immunotherapy-response analyses to operationalize these literature-motivated biological poles and were not selected or optimized on the basis of clinical outcomes. Gene-wise z-scores were calculated across samples and each module score was defined as the mean standardized expression of its component genes. The primary continuous immune axis was defined as the inflamed score minus the myeloid score. The checkpoint-top-30% subset was retained as the historical discovery entry rule because it was the subset used to construct the frozen compact classifier. In the original exploratory workflow, checkpoint-based entry windows spanning the top 20%, 25%, 30%, and 35% of the checkpoint distribution were compared, and the route-screening procedure included OS information. Accordingly, we do not interpret the top-30% threshold as a uniquely optimal or biologically privileged cutoff. To test sensitivity to the exact historical checkpoint cutoff without using outcome information, we repeated immune-active entry at the top 20%, 25%, 30%, and 35% of the checkpoint-score distribution. Within each entry window, the continuous inflamed-minus-myeloid axis and median-based operational end-group labels were recalculated using the same fixed modules. We quantified sample-set overlap with the historical checkpoint-top-30% subset, operational-label concordance among overlapping samples, and Hedges’ g for checkpoint, inflamed, myeloid, antigen-presentation, and T-cell-support programs. Survival outcomes were not used in this sensitivity analysis. Sensitivity analyses additionally evaluated top-30% entry based on an immune composite, the inflamed module, cytolytic activity, T-cell support, CD8 expression, immune infiltration, and a T-cell-inflamed gene-expression profile. For each entry definition, sample overlap and preservation of IFNγ-versus-myeloid biological polarity were assessed. Within the locked checkpoint-high subset, the continuous immune axis was evaluated using one-, two-, and three-component Gaussian mixture models with the Akaike information criterion (AIC) and Bayesian information criterion (BIC), together with Ashman’s D, kernel-density mode counting, silhouette coefficients, and bootstrap stability analysis. Gaussian-mixture fitting used scikit-learn GaussianMixture with n_init = 20 and random_state = 20 to reduce sensitivity to initialization. Initialization robustness was assessed by repeating the full-sample one-, two-, and three-component comparison across random_state values 0–99, each with n_init = 20, and recording the BIC-optimal component count. Finite-mixture models were used only as distributional diagnostics and were not interpreted as evidence that the fitted component count represented a corresponding number of biological classes. The two-component fit was examined as a stress test of the historical two-end labeling scheme, whereas kernel-density mode counting was used to assess empirical multimodality. Kernel-density estimates were calculated using SciPy’s Gaussian kernel-density estimator with its default bandwidth rule on a 1,024-point grid spanning 0.5 standard deviations beyond the observed minimum and maximum; local maxima with prominence ≥10% of the maximum estimated density were counted as prominent modes. Bootstrap stability was assessed in 1,000 nonparametric resamples using fixed seed 20260808. In each resample, the one- and two-component mixtures were refitted using n_init = 20 and random_state = 20, the one-versus-two-component BIC contrast, two-component Ashman’s D, and prominent-mode count were recalculated, and the bootstrap two-component model was projected back to the original observations on the bootstrap-standardized scale to calculate the adjusted Rand index relative to the full-sample two-component assignment. The historical median split was used only to generate operational end-group labels for panel development and descriptive comparisons and was not treated as a fitted biological boundary. The continuous immune axis, rather than any fitted mixture-component count or operational group assignment, was treated as the primary biological representation throughout the analysis.

### Compact transfer-panel development and external transfer

The compact panel was derived in the historical classifier-building workflow from a biologically restricted candidate pool comprising genes in the prespecified checkpoint, inflamed, myeloid, antigen-presentation, and T-cell-support modules. Candidate genes were ranked once in the full historical TCGA discovery subset by the absolute difference in mean standardized expression between the operational IFNγ-responsive-associated and myeloid-suppressive-associated end groups, while retaining representation from both biological directions. Fixed candidate panel sizes from 10 through 16 genes were then compared using fivefold stratified nearest-centroid cross-validation. Because the feature-ranking step preceded rather than being repeated within each cross-validation training fold, these cross-validation results are treated only as a historical panel-size screening heuristic and are not interpreted as unbiased estimates of out-of-sample classifier performance. The candidate panel maximizing the historical screening criterion within the prespecified 10–16-gene range was retained for deployment. This procedure yielded a locked 10-gene panel comprising five genes originally selected from the IFNγ-associated direction (*CXCL9, B2M, IFNG, CTLA4,* and *PRF1*) and five genes originally selected from the myeloid-associated direction (*VEGFA, TREM2, TGFB1, APOE,* and *SPP1*). These historical selection directions refer to the standardized-expression screen used for panel construction; the final frozen template-contrast weights were recalculated after stability-enriched within-sample rank recentering and therefore need not preserve the same sign for every individual gene. Panels smaller than 10 genes were not evaluated, and neither the panel size nor the exact feature set is claimed to be globally optimal, minimally sufficient, or superior to simpler immune signatures. After panel selection, stability-enriched re-centering was performed only within the already labeled TCGA immune-active subset and did not reopen gene selection or relabel tumors. Final cross-platform deployment used within-sample panel ranks and the frozen template-contrast rule with fixed weights and class-specific uncertainty thresholds. Importantly, the biological discovery axis and the frozen transfer score are related but not numerically identical quantities. The discovery axis is the continuous inflamed-minus-myeloid module difference, whereas the frozen template-contrast score is a signed 10-gene rank-based deployment output constructed using the operational end-group labels and oriented so that higher values indicate the IFNγ-associated direction. The template-contrast score was retained continuously for cross-cohort association analyses; fixed class-specific thresholds were applied only when categorical operational labels were required, and samples falling between the two thresholds were assigned to an intermediate group. Thus, the thresholded operational labels are discretized summaries of a continuous transfer score rather than evidence that the underlying biology is binary. External datasets were not used to reselect genes, change score direction, optimize thresholds, or choose the deployment method. For the seven prespecified GSE14520 external-polarity contrasts (checkpoint, inflamed, antigen presentation, T-cell support, myeloid, suppressive core, and inflamed-minus-myeloid), two-sided Mann–Whitney U tests compared the non-intermediate IFNγ-associated and myeloid-associated groups, and the resulting P values were adjusted together using the Benjamini–Hochberg procedure. For treatment cohorts with incomplete panel coverage, gene availability was reported explicitly; GSE140901 contained 9 of 10 genes and lacked *B2M*, and a TCGA *B2M*-dropout sensitivity analysis was performed.

### Single-cell, spatial, proteomic, and phosphoproteomic analyses

GSE149614 was reanalyzed from the normalized single-cell matrix with exact cell-identifier matching to metadata. The frozen 10-gene template-contrast score was calculated primarily from sample-level and sample-by-cell-type pseudobulk profiles, with per-cell scores retained for descriptive localization. Because multiple anatomical specimens were available from some patients, sample-level pseudobulk aggregation reduces cell-level pseudoreplication but does not render all anatomical-sample analyses patient-independent. Accordingly, correlations across anatomical samples were treated as descriptive sample-level associations, whereas paired tumor-versus-non-tumor comparisons were evaluated within patient pairs. Strict non-overlap programs were constructed after excluding every compact-panel gene and all genes used in the original checkpoint, inflamed, and myeloid state definitions; these included strict non-overlap antigen-processing, T-cell-effector, macrophage, fibroblast/ECM, and hypoxia programs. These revision-stage strict non-overlap programs were introduced specifically as sensitivity analyses to test whether the observed biological polarity persisted after removal of all compact-panel and original state-defining genes. They were not used to define the discovery axis, assign operational labels, select compact-panel genes, or tune the frozen transfer score; accordingly, they are interpreted as non-overlap sensitivity support rather than as independent de novo biological discovery. For GSE149614 sample-level strict non-overlap analyses, we restricted inference to the 13 tumor-related anatomical samples (primary HCC, portal-vein tumor thrombus, or metastatic lymph-node specimens) and excluded non-tumor liver samples. For each of the five strict non-overlap programs, a two-sided Spearman correlation was calculated against the frozen 10-gene template-contrast score across these 13 anatomical samples. Benjamini–Hochberg correction was applied jointly to the five program-level correlation P values; this five-test family yielded an adjusted P value of 0.0133 for the antigen-processing association. Existing CellChat and focused NicheNet analyses were retained as communication-pathway characterization rather than independent validation. GSE245908 was analyzed in two analyzable Visium sections. Spot-level inflamed-minus-myeloid spatial-ecology scores, reference-bridge correlations, neighborhood enrichment, Ripley-based summaries, and nearest distances from IFNγ-high to myeloid-high spots were evaluated to quantify local spatial organization. For endpoint-based neighborhood summaries, IFNγ-high and myeloid-high spots were defined by the frozen spatial-ecology labels generated separately within each section as the upper and lower tertiles, respectively, of state_axis_ecology; middle-tertile spots were retained as intermediate. The separately derived state_axis_bridge was used only for reference-bridge correlation and annotation and was not used to redefine the spatial endpoint labels. To define a local neighborhood radius without tuning to the observed IFNγ-high/myeloid-high proximity results, we used the native Visium array geometry. Direct lattice-neighbor spot pairs were identified from the Visium array coordinates using the three forward hexagonal-neighbor offsets (0, + 2), (+1,-1), and (+1, + 1), and their full-resolution image center-to-center distances were calculated from the source tissue-position files. Across the two analyzed GSE245908 sections, the largest direct-neighbor center distance was 221.777 pixels; we therefore used its ceiling, 222 full-resolution pixels, as a radius corresponding to one direct Visium lattice-neighbor center-to-center step. This radius was defined from platform geometry rather than from the IFNγ-high/myeloid-high spot labels or the observed IFNγ-high-to-myeloid-high distance distribution and is distinct from the spot diameter (~143 full-resolution pixels in these sections). To test whether the observed immediate-neighborhood cross-end contact fraction differed from that expected from endpoint abundance alone, we performed 10,000 count-preserving within-section label permutations using fixed seed 20260809. In each permutation, the frozen IFNγ-high, myeloid-high, and intermediate labels were randomly reassigned over the fixed spot coordinates while preserving the number of spots in each category, and the fraction of IFNγ-high spots with at least one myeloid-high spot within 222 pixels was recalculated. Empirical tail probabilities used a + 1 correction, and two-sided empirical P values were calculated as twice the smaller of the lower- and upper-tail probabilities, capped at 1. This permutation null preserves endpoint abundance but does not preserve higher-order spatial autocorrelation or tissue architecture and was therefore interpreted only as an abundance-matched random-label reference rather than as a complete null model of tissue architecture. For visualization, representative proximal mixed neighborhoods were selected algorithmically as IFNγ-high spots with the largest numbers of myeloid-high neighbors within the analysis radius; highlighted regions were used only for illustration and not for statistical inference.

PDC000198 and PDC000199 were analyzed as paired cross-modal molecular layers from 165 HCC and adjacent non-tumoral tissue pairs. PDC000198 contained 6 of the 10 compact-panel proteins and was therefore used only for an exploratory partial-panel protein-axis projection, together with strict non-overlap protein programs. For PDC000199, RefSeq protein accessions were mapped to human gene symbols using NCBI gene-product records; 98.6% of phosphosites were mapped, and multiple phosphosites per gene were summarized by gene-level median phosphorylation. Because only 3 of the 10 compact-panel genes were represented at the phosphoprotein level, PDC000199 was not treated as classifier validation. Instead, prespecified phosphorylation programs, including SPP1-integrin-FAK, myeloid-receptor, fibroblast/ECM, hypoxia, IFNG-JAK-STAT, VEGF-PI3K-AKT-MAPK, MIF-CD74-CXCR4, and ECM-FAK-SRC, were related to the separately measured PDC000198 partial protein axis.

### Treatment-program derivation and untreated-HCC projection

GSE206325 was used only to construct an exploratory treatment-associated transcriptional bridge. Source-study response annotations were linked to biological sample identifiers, and cells with mappable sample and response labels were summed within biological sample to generate sample-level pseudobulk counts, followed by library-size normalization to counts per million and log1p transformation. This yielded 92 responder-labeled and 176 non-responder-labeled pseudobulk profiles. Because the source resource contains biological specimens collected at different treatment time points and may therefore contribute more than one profile from the same patient, these 268 profiles were not treated as independent patient-level observations. The profile-level contrast was used only for descriptive ranking of treatment-associated genes; no inferential P values, confidence intervals, patient-level response estimates, or predictive-performance measures were calculated from this contrast. For each gene, the descriptive responder-associated effect was defined as the difference in mean pseudobulk expression between responder-labeled and non-responder-labeled profiles, and the 50 genes with the largest positive and negative effects were frozen as the responder-up and responder-down programs, respectively. In untreated TCGA-LIHC and GSE14520, expression values were converted to within-sample percentile ranks, each program score was defined as the mean rank of the available program genes, and the responder axis was defined as responder-up score minus responder-down score. These projected scores were summarized descriptively within the previously assigned operational IFNγ-responsive-associated and myeloid-suppressive-associated groups. Because the projection cohorts did not provide corresponding immunotherapy-response outcomes, the projection was interpreted only as transcriptional similarity to GSE206325 treatment-associated programs and was not used to estimate or validate clinical response prediction.

### Direct HCC immunotherapy-cohort evaluation and meta-analysis

The previously frozen 10-gene template-contrast score was then applied directly, without retraining, to four public HCC immunotherapy cohorts: GSE140901, GSE215011, GSE279750, and GSE202069. For GSE202069, all 17 anti-PD-1-treated patients with available Response Evaluation Criteria in Solid Tumors (RECIST) response in the source clinical table were included. Cohort-specific responder definitions were retained. Continuous-score response associations within each treated cohort were summarized using the area under the receiver operating characteristic curve (AUC) with bootstrap 95% confidence intervals, Mann–Whitney tests, Hedges’ g, and odds ratios per one-standard-deviation increase where estimable. These metrics were used descriptively to quantify within-cohort response association and discrimination and were not interpreted as validated predictive performance. HCC cohort effects were pooled separately for Hedges’ g and log odds ratios per one-standard-deviation increase using random-effects models; AUCs were not pooled.

Exploratory GSE202069 survival associations were estimated in the same 17 treated patients using separate univariable Cox proportional-hazards models for PFS and OS, with the frozen continuous template-contrast score standardized within the treated subset. We report the log-hazard coefficient (β), its standard error, Wald z statistic, hazard ratio per one-standard-deviation increase, 95% confidence interval, and two-sided Wald P value. These survival analyses were descriptive, were not multiplicity-adjusted, and were not used to support prognostic, predictive, or treatment-specific claims.

For Hedges’ g, sampling variance was estimated as (nR + nNR)/(nR × nNR) + g²/[2(nR + nNR − 3.94)], and random-effects pooling used a DerSimonian–Laird method-of-moments estimate of τ². Given the small number of available HCC treatment cohorts, the primary Hedges’ g random-effects synthesis was accompanied by a small-k sensitivity analysis using restricted maximum likelihood estimation of τ² and a Hartung–Knapp adjustment for the pooled confidence interval. This sensitivity analysis was used to assess the robustness of the inferential conclusion rather than to select the most favorable pooled estimate. Simple comparator scores (*IFNG* alone, *CXCL9* alone, cytolytic activity, checkpoint, inflamed, myeloid-inverse, and inflamed-minus-myeloid scores) were evaluated as exploratory benchmarks. Comparator scores used the original prespecified discovery-module definitions. Cytolytic activity was defined from *GZMA* and *PRF1*; myeloid-inverse was the negative of the prespecified myeloid score; and inflamed-minus-myeloid used the same fixed inflamed and myeloid modules. Gene-wise module standardization was performed within each source expression matrix before response-subset extraction; for GSE202069, module z-scores were therefore computed in the full source matrix before restriction to the 17 anti-PD-1-treated patients. Comparator AUC 95% CIs reported in Table S4E in S2 File were obtained from 5,000 nonparametric bootstrap resamples, discarding resamples containing a single response class.

Cross-cancer treated cohorts were retained only as secondary contextual analyses and were not used to support HCC-specific clinical predictive claims. Because none of the four HCC treatment cohorts included a matched non-ICI comparator arm suitable for biomarker-by-treatment interaction testing, these analyses were interpreted as response associations within treated HCC rather than evidence of treatment-specific predictive value.

### Upstream multi-omic anchoring

Upstream TCGA-LIHC anchoring remained restricted to prespecified expression axes, candidate-gene contrasts, mutation enrichment, gene-level copy-number support, and methylation anchors. For the expression-space anchoring, values were calculated on the source TCGA-LIHC expression scale: each prespecified expression axis was summarized as the mean expression of its available component genes within each operational end group, and candidate genes were summarized by group-specific mean expression. For visualization, the IFNγ-associated minus myeloid-associated group mean difference was used to indicate direction only; because these features are represented on different source-expression scales, the magnitudes are not interpreted as standardized effect sizes or compared quantitatively across features.

All tests were two-sided, false-discovery-rate (FDR) correction was applied where multiple prespecified features were tested, and confidence intervals and effect sizes were prioritized over dichotomous significance claims.

### Reproducibility safeguards and claim boundaries

The inflamed and myeloid modules and the direction of the continuous inflamed-minus-myeloid axis were defined before survival or immunotherapy-response analyses and were not selected using clinical outcomes. The historical checkpoint-top-30% plus axis-median operational route, by contrast, was selected during exploratory TCGA screening that included OS information; therefore, TCGA survival is not used as evidence validating the biological framework. The biological interpretation is instead supported by outcome-independent checkpoint-threshold and alternative-entry sensitivity analyses, direct continuity/modality analyses, frozen external transfer, strict non-overlap transcriptomic support, complementary single-cell and spatial characterization, and cross-modal proteomic/phosphoproteomic support. For all external treatment analyses, the compact 10-gene panel, score direction, template-contrast weights, uncertainty thresholds, and deployment strategy were frozen before treatment outcomes were evaluated. External HCC cohorts were used to assess transferability rather than to reselect genes or redefine the axis. Evidence that reused state-defining genes was described as biological characterization. Strict non-overlap transcriptomic programs were treated as support based on gene sets that excluded the compact-panel and original state-defining genes, sample-aware single-cell and spatial analyses as complementary localization and ecological characterization, and paired proteomic/phosphoproteomic layers as cross-modal biological support. Because no new clinical samples or functional experiments were generated and the available HCC immunotherapy cohorts were small and heterogeneous, treatment-cohort analyses are explicitly bounded as exploratory association analyses rather than validation of a clinically deployable response predictor.

## Results

### Immune-active HCC is organized along a structured IFNγ-to-myeloid immune continuum

We first assessed the internal structure of the 112 checkpoint-high TCGA-LIHC tumors defined by the historical checkpoint-top-30% entry rule. BIC favored the three-component Gaussian-mixture model (290.47) over the two- (304.43) and one-component (327.28) models, and this ordering was stable across initialization-sensitivity analyses. However, the observed kernel-density curve contained one prominent mode, none of 1,000 bootstrap resamples showed two or more prominent modes, and the median bootstrap adjusted Rand index for the two-component assignment was 0.504. Although mixture models captured non-Gaussian internal structure, the absence of robust empirical multimodality and incomplete categorical stability did not support naturally discrete biological populations. We therefore used the continuous axis as the primary representation and retained the median split only for operational end-group summaries, yielding 56 IFNγ-responsive-associated and 56 myeloid-suppressive-associated tumors (Fig 2A,B; Fig S1C in S1 File; Table S1A in S2 File).

**Fig 2:**
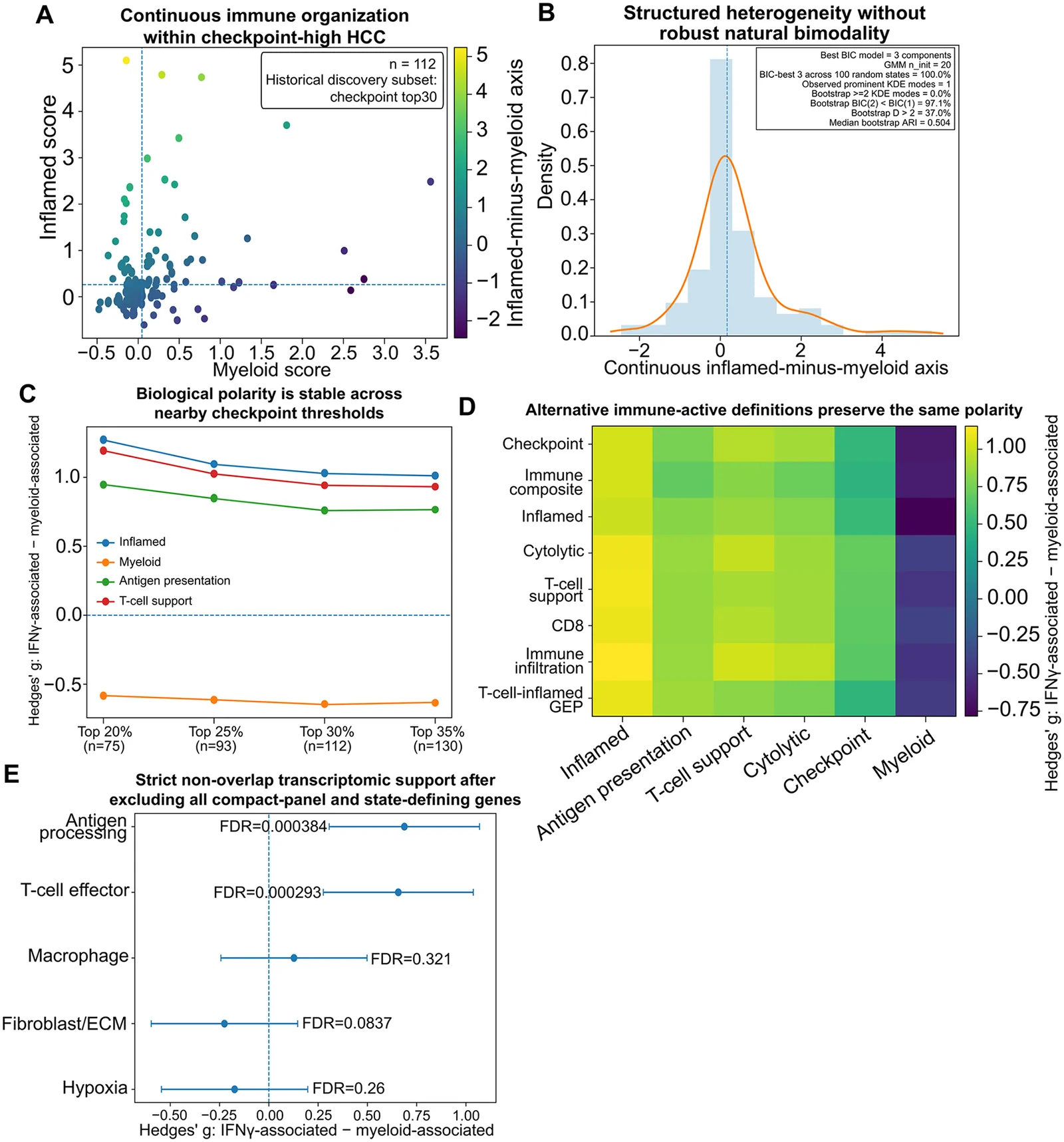
Discovery, sensitivity, and continuity analyses of immune-active HCC in TCGA-LIHC. (A) Inflamed-versus-myeloid immune space for the 112 tumors in the historical checkpoint-top-30% discovery subset, colored by the continuous inflamed-minus-myeloid axis. (B) Distributional diagnostics: although Gaussian-mixture BIC favored a multicomponent approximation, the observed kernel-density curve contained one prominent mode, no bootstrap resample showed ≥2 prominent modes, and two-component assignments were only moderately stable (median ARI = 0.504). (C) Outcome-independent checkpoint-entry sensitivity across top-20% to top-35% windows. (D) Biological polarity across eight alternative immune-active entry definitions. (E) Strict non-overlap bulk-program effects after excluding compact-panel and original state-defining genes. These analyses support use of the continuous axis as the primary representation.

Outcome-independent checkpoint-threshold sensitivity further showed that the principal biological polarity did not depend on the exact historical 30% cutoff. Checkpoint-high entry at the top 20%, 25%, 30%, and 35% selected 75, 93, 112, and 130 tumors, respectively. Relative to the checkpoint-top-30% subset, sample-set Jaccard indices were 0.670, 0.830, 1.000, and 0.862, while operational-label concordance among overlapping samples was 93.3%, 93.5%, 100%, and 97.3%. Across the four windows, Hedges’ g remained positive for inflamed (1.01–1.27), antigen-presentation (0.76–0.95), and T-cell-support programs (0.93–1.19) and negative for the myeloid program (−0.65 to −0.58). These findings indicate that the principal biological polarity is not contingent on the exact checkpoint-top-30% cutoff (Fig 2C; Fig S1A,D in S1 File; Table S1B in S2 File).

The principal biological polarity was robust to alternative definitions of immune-active entry. Top-30% selection based on immune composite, inflamed, cytolytic, T-cell-support, CD8, immune-infiltration, and T-cell-inflamed-GEP scores showed only partial sample overlap with the historical checkpoint-top-30% subset (Jaccard 0.534–0.647), yet each definition preserved higher inflamed, antigen-presentation, T-cell-support, and cytolytic programs toward the IFNγ-associated end and higher myeloid scores toward the opposing end (Fig 2D; Fig S1B in S1 File; Table S1C in S2 File). To reduce circularity, we also repeated downstream bulk characterization with strict non-overlap signatures that excluded all compact-panel and state-defining genes. Strict non-overlap antigen-processing and T-cell-effector programs remained higher in the IFNγ-associated group (Hedges’ g = 0.688 and 0.657; FDR = 3.84 × 10^-4^ and 2.93 × 10^-4^, respectively), whereas the strict non-overlap macrophage program was not significant and fibroblast/ECM showed only a directional myeloid-side trend (Fig 2E; Table S1D in S2 File). Thus, these analyses support coordinated axis-associated immune ecologies while avoiding the claim that the two operational labels are naturally discrete populations.

### A locked 10-gene template-contrast rule provides a portable continuous proxy for axis position across HCC cohorts

Within the historical 10–16-gene panel-size screen, the 10-gene candidate achieved the highest prespecified engineering score and was retained (Fig 3A; section S2D of S1 Table in S2 File). The fixed panel comprised five genes from the historical IFNγ-associated selection direction (*CXCL9*, *B2M*, *IFNG*, *CTLA4*, and *PRF1*) and five from the historical myeloid-associated direction (*VEGFA*, *TREM2, TGFB1*, *APOE*, and *SPP1*) (Fig 3B; sections S2A and S2B of S1 Table in S2 File). The locked rule produced a continuous signed template-contrast score, with fixed thresholds used only for optional operational labels and intermediate samples retained. Accordingly, the 10-gene panel is interpreted as a compact deployment representation of the continuous biological axis rather than as a uniquely optimal feature set or evidence of superiority over simpler immune signatures.

**Fig 3:**
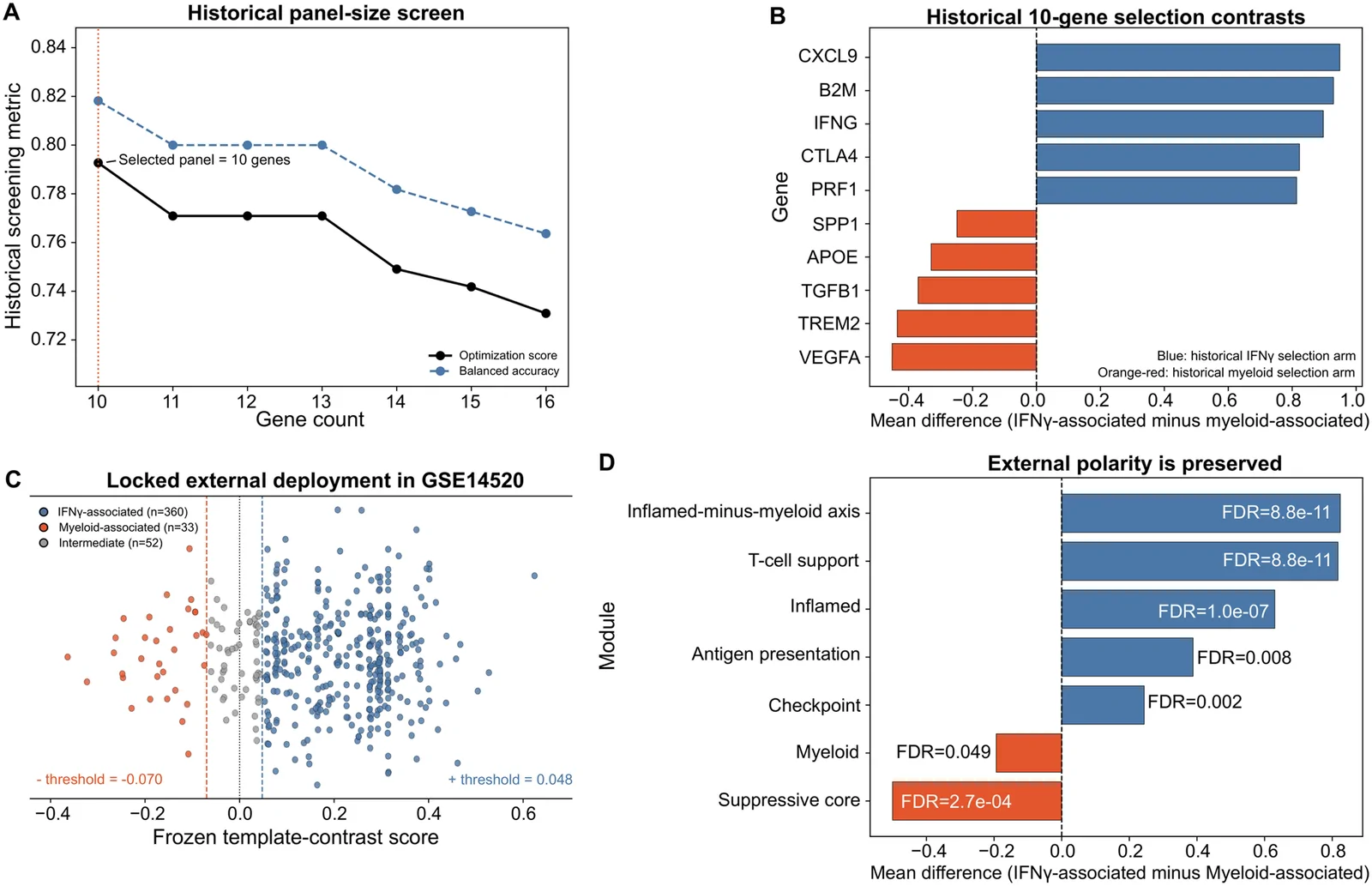
A locked 10-gene template-contrast rule provides a portable continuous proxy for axis position across HCC cohorts. (A) Historical panel-size screen across 10–16-gene candidates; the internal metric is shown only as a development-stage engineering comparison. (B) Standardized-expression mean differences for the 10 genes retained during historical panel construction; colors indicate the historical selection direction. (C) Continuous frozen template-contrast scores in GSE14520 with fixed thresholds defining optional IFNγ-associated, myeloid-associated, and intermediate labels. (D) Prespecified external biological-polarity contrasts in GSE14520. The transfer score remains continuous and is not presented as a validated clinical response predictor.

After fixed probe-to-symbol remapping, GSE14520 retained all 10 panel genes and 445 evaluable expression profiles. The frozen transfer rule assigned 360 tumors (80.9%) to the IFNγ-responsive-associated side, 33 (7.4%) to the myeloid-suppressive-associated side, and 52 (11.7%) to the intermediate group. The unequal external class frequencies reinforce that these labels are operational projections of a continuous biological axis rather than evidence of two equally prevalent natural populations (Fig 3C; Table S2C in S2 File).

In non-intermediate GSE14520 tumors, all seven prespecified external-polarity contrasts were oriented as expected after Benjamini–Hochberg correction: checkpoint, inflamed, antigen-presentation, T-cell-support, and inflamed-minus-myeloid signals were higher on the IFNγ-associated side, whereas myeloid and suppressive-core signals were higher on the myeloid-associated side (FDR range, 8.78 × 10^-11^ to 0.0486; Fig 3D). Recurrence-free survival (RFS) was non-significant and OS showed only directional support in GSE14520; donor-collapsed ICGC-LIRI-JP provided concordant but non-significant survival context. These findings demonstrate cross-platform deployment of the frozen score with preservation of its expected biological orientation. Because several of the external polarity summaries evaluate biology related to the original axis definition, they are interpreted as transferability and consistency evidence rather than as independent biological validation or prognostic performance (Fig 3D; Fig S2A-C in S1 File; Table S2E in S2 File).

### Single-cell, spatial, proteomic, and phosphoproteomic data provide complementary ecological characterization and cross-modal support

Across 71,915 GSE149614 cells from 21 samples, 10 patients, and six major cell types, the frozen 10-gene template-contrast score showed reproducible sample- and compartment-level organization. Across sample-by-cell-type pseudobulk profiles, T/NK cells had the highest mean template-contrast score (+0.107) and myeloid cells the lowest (−0.188), consistent with localization of opposing immune tendencies to distinct cellular compartments (Fig 4A; Table S3B in S2 File). In eight paired primary-HCC/non-tumor comparisons, the sample-level pseudobulk template-contrast score shifted toward the myeloid-oriented direction in tumor tissue (median tumor-minus-non-tumor change = −0.394; paired Wilcoxon P = 0.0078; Fig 4B; Table S3A in S2 File). Cell-composition analyses showed a positive association between the sample-level template-contrast score and T/NK fraction (Spearman rho = 0.629, FDR = 0.0068) and an inverse association with hepatocyte fraction (rho = −0.732, FDR = 9.58 × 10^-4^; Fig S3A in S1 File). In strict non-overlap analyses of 13 tumor-related anatomical samples, the antigen-processing program correlated positively with the frozen 10-gene template-contrast score (Spearman rho = 0.758; raw P = 0.00267; Benjamini–Hochberg FDR = 0.0133 across the five strict non-overlap programs; Fig 4C; Table S3C in S2 File). Because these anatomical samples include repeated specimens from some patients, these statistics are interpreted as supportive sample-level associations rather than independent patient-level validation. The cell-type pseudobulk analysis is therefore interpreted primarily as biological localization, with the continuous antigen-processing association providing transcriptomic support from a strict non-overlap gene set for the IFNγ-oriented tendency of the axis.

**Fig 4:**
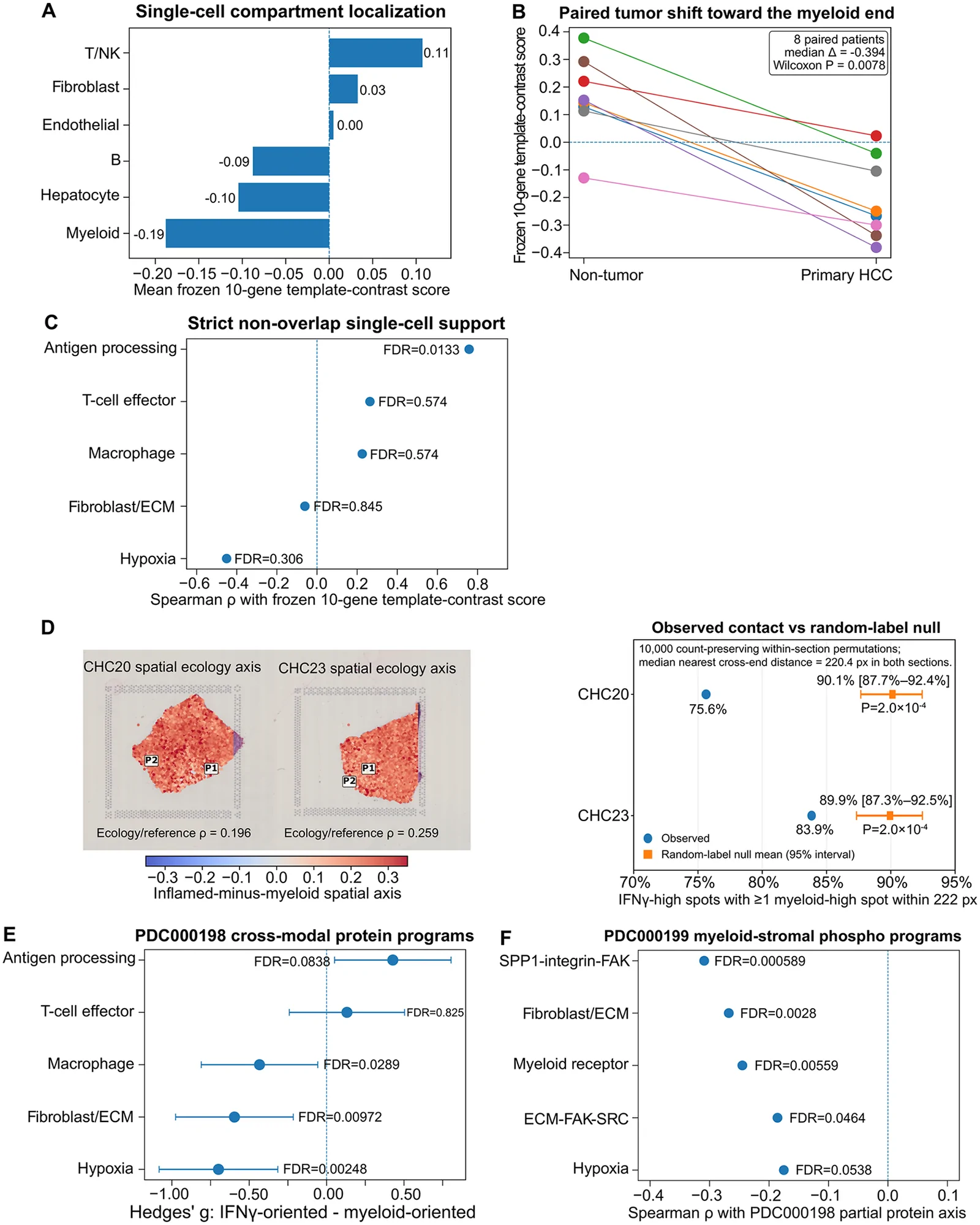
Single-cell, spatial, proteomic, and phosphoproteomic evidence along the immune continuum. (A) Mean frozen 10-gene template-contrast scores across GSE149614 sample-by-cell-type pseudobulk profiles. (B) Paired primary-HCC versus non-tumor sample-level scores in eight patients. (C) Correlations of strict non-overlap single-cell programs with the frozen template-contrast score. (D) GSE245908 spatial-ecology maps for CHC20 and CHC23 and section-wide comparison of observed IFNγ-high–to–myeloid-high contact fractions with the 10,000-permutation random-label null within the geometry-defined 222-pixel neighborhood. P1/P2 mark illustrative proximal mixed neighborhoods. (E) PDC000198 strict non-overlap protein-program effects; negative Hedges’ g indicates stronger signal toward the myeloid-oriented end. (F) PDC000199 phosphorylation-program correlations with the PDC000198 partial protein axis; negative correlations indicate stronger myeloid-oriented phosphorylation. PDC000199 cross-modal results are not interpreted as direct validation of the full 10-gene classifier.

Supportive CellChat and focused NicheNet analyses further suggested an SPP1/MIF-centered remodeling niche, highlighting SPP1-CD44/integrin, MIF-CD74/CXCR4/CD44, FN1-CD44, and VEGF-receptor-linked communication on the myeloid-oriented side (Fig S3B in S1 File). Because these inference-based analyses partly reuse biology related to the state definition, we treat them as hypothesis-generating communication-pathway characterization rather than independent validation or functional evidence. The strict non-overlap transcriptomic analyses mainly provide support from gene sets non-overlapping with the compact panel and original state-defining genes for the IFNγ-oriented end, whereas stronger cross-modal support for the myeloid-oriented end comes from the paired proteomic and phosphoproteomic analyses described below.

Spatial analyses provided complementary localization and neighborhood-organization evidence. In the two analyzable GSE245908 sections, the ecology-axis/reference-bridge Spearman correlations were modest (ρ = 0.196 for CHC20 and ρ = 0.259 for CHC23; Fig 4D; Fig S3C,D in S1 File; Table S3D in S2 File). Using the frozen within-section spatial-ecology endpoint labels, 570/754 (75.6%) IFNγ-high spots in CHC20 and 556/663 (83.9%) in CHC23 had at least one myeloid-high spot within the geometry-derived 222-pixel neighborhood radius, and the median nearest cross-end distance was 220.4 pixels in both sections. Despite these high absolute contact fractions, 10,000 count-preserving within-section label permutations yielded higher abundance-matched expectations: 90.1% (95% permutation interval, 87.7–92.4%) for CHC20 and 89.9% (87.3–92.5%) for CHC23. The observed contact fraction was lower than the random-label expectation in both sections (two-sided empirical P = 2.0 × 10 − 4 for each; Fig 4D). Thus, the spatial data support frequent local proximity of the opposing programs in absolute terms but reduced immediate cross-end mixing relative to an abundance-matched random-label null, rather than preferential co-localization. The paired proteomic and phosphoproteomic datasets supplied stronger cross-modal support for myeloid-stromal remodeling. In PDC000198, tumors at the myeloid-oriented end of the partial protein axis showed higher strict non-overlap macrophage, fibroblast/ECM, and hypoxia programs (FDR = 0.0289, 0.0097, and 0.0025, respectively; Fig 4E; Table S3E in S2 File). In PDC000199, across the same 165 tumor samples, SPP1-integrin-FAK, fibroblast/ECM, myeloid-receptor, and ECM-FAK-SRC phosphorylation programs were inversely associated with the PDC000198 partial protein axis (Spearman rho = −0.309, −0.268, −0.245, and −0.186; FDR = 5.89 × 10^-4, 0.0028, 0.0056, and 0.0464; Fig 4F). Extreme-group analyses showed the same myeloid-side enrichment for SPP1-integrin-FAK, myeloid-receptor, fibroblast/ECM, and hypoxia phosphorylation (FDR ≤ 0.0454; Table S3F in S2 File). Protein-abundance and gene-level phosphorylation tumor-minus-paratumor changes were strongly concordant across 4,889 overlapping genes (Spearman rho = 0.724; Table S3G in S2 File). Because PDC000199 contained only 3 of the 10 compact-panel genes at the phosphoprotein level, these data are interpreted as cross-modal molecular concordance with the myeloid-oriented ecology rather than as classifier validation or evidence of a causal mechanism.

### Direct HCC treatment-cohort evaluation does not establish clinical response prediction

Projection of the fixed GSE206325 responder programs into untreated TCGA-LIHC and GSE14520 provided an HCC-specific biological bridge to anti-PD-1-associated transcription (Fig 5A,B; Table S4G in S2 File). In both untreated cohorts, the IFNγ-associated group showed higher mean responder-up and responder-axis scores than the myeloid-associated group. This directional pattern is interpreted only as transcriptional resemblance to the GSE206325 responder-associated program. Because corresponding immunotherapy-response outcomes were unavailable in these projection cohorts, no response probability, discrimination, calibration, or clinical benefit was inferred from this analysis.

**Fig 5:**
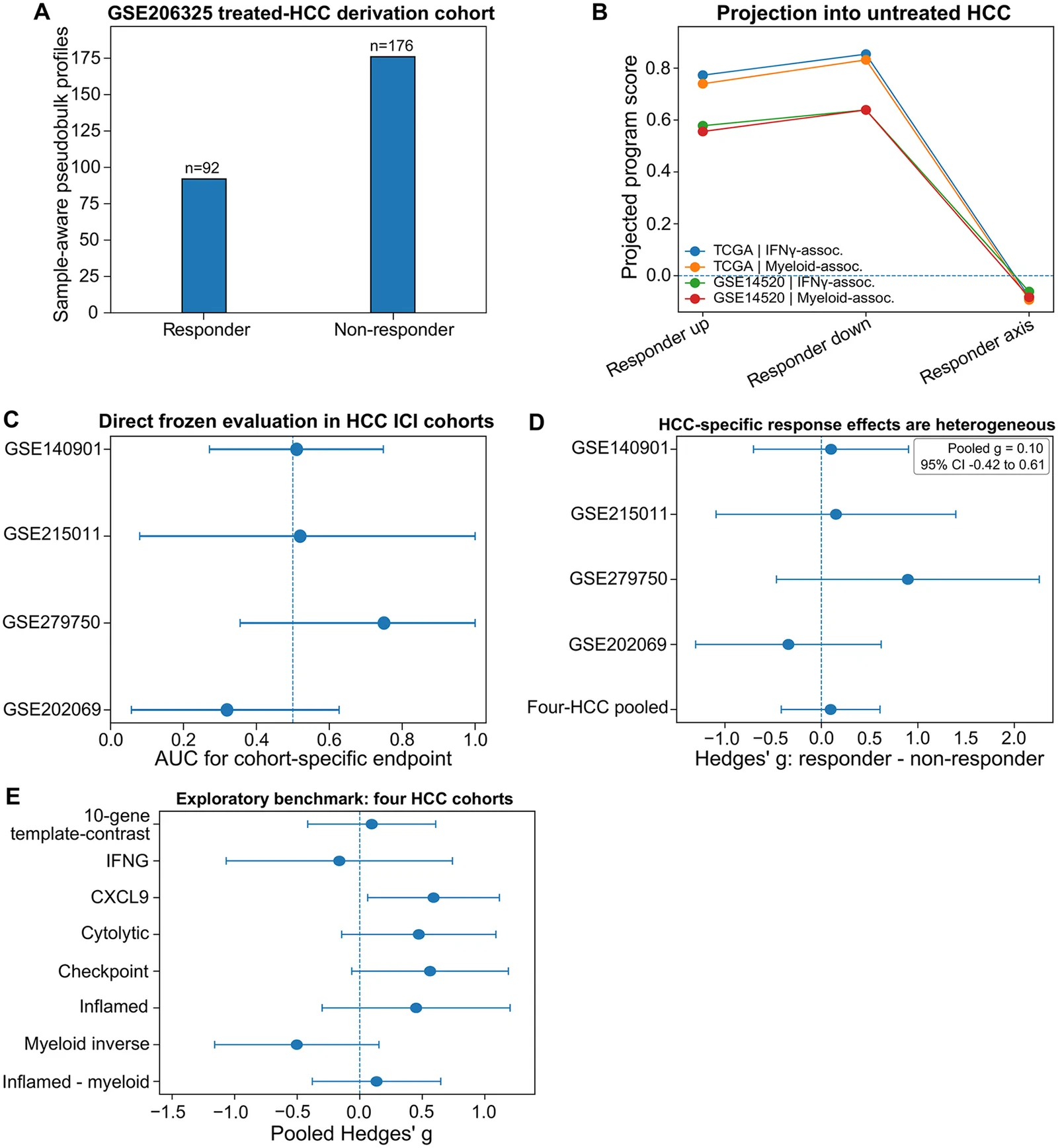
Exploratory treatment relevance and direct HCC immunotherapy evaluation. (A) GSE206325 responder- and non-responder-labeled sample-level pseudobulk profiles used to derive fixed responder-associated programs; counts denote profiles rather than independent patients. (B) Descriptive projection of the frozen responder-up and responder-down programs into untreated TCGA-LIHC and GSE14520; the responder axis represents transcriptional similarity, not response probability. (C) Cohort-specific AUCs after frozen application of the 10-gene template-contrast score to four public HCC immunotherapy cohorts. (D) Cohort-specific Hedges’ g values and the random-effects pooled estimate. (E) Exploratory pooled benchmark against seven simpler immune signatures. The treatment analyses are hypothesis-generating and do not establish a validated clinical response predictor.

We next applied the previously frozen 10-gene template-contrast score directly to four public HCC treatment cohorts without retraining or threshold optimization. GSE140901 (n = 24; clinical-benefit endpoint) yielded AUC = 0.510 and Hedges’ g = 0.100; GSE215011 (n = 10) yielded AUC = 0.520 and g = 0.151; GSE279750 (n = 10) yielded AUC = 0.750 and g = 0.896; and GSE202069 (n = 17 anti-PD-1-treated patients with response annotation) yielded AUC = 0.319 and g = −0.342. The corresponding responder/non-responder score distributions are shown in Fig S4A in S1 File, and the complete cohort-level direct-evaluation metrics are provided in Table S4A in S2 File. Exploratory univariable PFS and OS associations in the small GSE202069 treated subset are reported only as descriptive context; they were not multiplicity-adjusted and are not used to support prognostic, predictive, or treatment-specific claims (Table S4B in S2 File). Because the cohort-specific binary endpoints were not identical—GSE140901 used clinical benefit, whereas the other cohorts used response-based endpoints—the random-effects synthesis is interpreted as a descriptive summary of score–outcome association across treated HCC cohorts rather than as a harmonized estimate of one common objective-response endpoint. The cohort-specific AUC estimates are summarized in Fig 5C, whereas cohort-specific Hedges’ g values and the random-effects pooled estimate are shown in Fig 5D; corresponding odds-ratio and leave-one-cohort-out analyses are provided in Fig S4B,C in S1 File and Table S4A in S2 File. GSE140901 lacked *B2M* and therefore contained 9 of 10 panel genes; TCGA dropout sensitivity showed high continuous-score robustness (Pearson r = 0.994; 92.5% sign concordance) but lower three-class threshold concordance (84.1%), supporting caution in thresholded interpretation of that cohort (Fig S4D in S1 File; Table S4D in S2 File).

Exploratory benchmark analyses still did not show clear superiority of the frozen 10-gene template-contrast score over simpler immune scores: across the same four HCC immunotherapy cohorts, *CXCL9* alone produced the strongest pooled positive signal (Hedges’ g = 0.59, 95% CI 0.06–1.12), whereas the frozen 10-gene template-contrast score remained smaller (g = 0.10, 95% CI −0.42 to 0.61) (Fig 5E; Fig S4E in S1 File; Table S4C,E in S2 File). A small-k sensitivity analysis using REML and Hartung–Knapp produced the same pooled point estimate with a wider confidence interval (Hedges’ g = 0.10, 95% CI −0.60 to 0.80), reinforcing the conclusion that the available HCC treatment cohorts do not establish a precise positive response association (Table S4H in S2 File). Together, these analyses show that the framework can be deployed without outcome-based retuning, but they do not support claims of validated predictive performance, added clinical value, or superiority over simpler immune signatures. Supportive paired analyses in GSE285963 and GSE202069 are provided only as secondary context and were not included in the four-HCC response meta-analysis (Fig S4F in S1 File; Table S4F in S2 File).

### Upstream multi-omic anchoring of axis-associated immune tendencies

Prespecified upstream analyses in TCGA-LIHC showed that the operational axis-associated labels were linked to coherent molecular contexts. The IFNγ-associated side showed higher inflammatory-core and checkpoint-associated signals, whereas the myeloid-associated side showed higher myeloid-suppressive-core and suppressive-panel signals. These analyses provide contextual molecular anchoring of the two axis tendencies rather than evidence that upstream genomic alterations create two naturally discrete immune classes (Fig 6A; Fig S5A-F in S1 File; Table S5 in S2 File).

**Fig 6:**
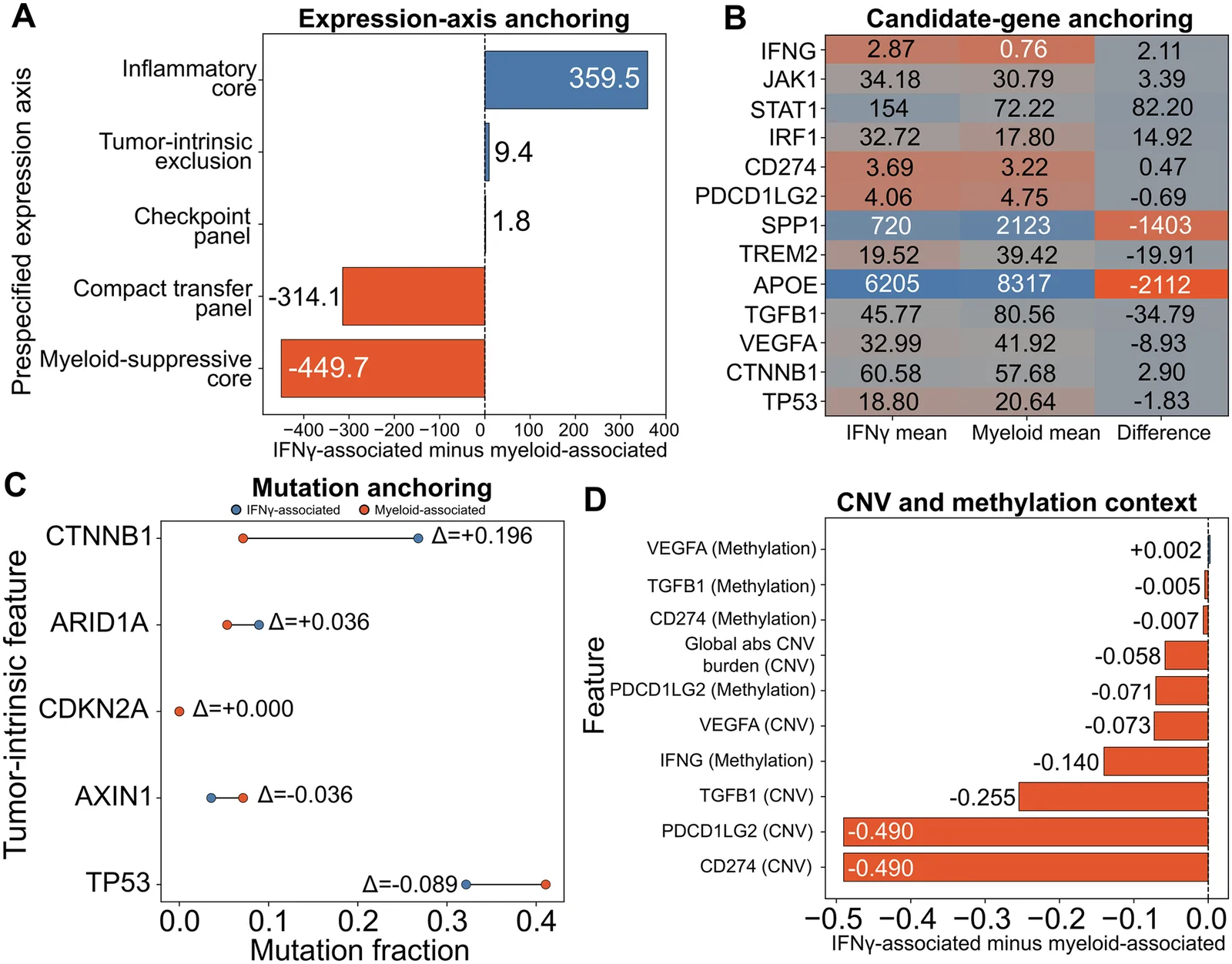
Upstream multi-omic anchoring of axis-associated immune tendencies. (A) Prespecified expression-space anchoring in TCGA-LIHC under the operational axis-associated labels. Bars show source-scale group mean differences (IFNγ-associated minus myeloid-associated) for the indicated prespecified gene sets; values are used to indicate direction only and are not standardized effect sizes, so magnitudes should not be compared across features. (B) Candidate-gene anchoring showing source-scale group means and IFNγ-associated minus myeloid-associated differences for prespecified inflammatory, suppressive-remodeling, checkpoint-axis, and tumor-associated markers. (C) Mutation anchoring for prespecified tumor-associated genes, shown as relative asymmetries rather than state-defining events. (D) Copy-number and methylation context for the myeloid-oriented end, including global absolute copy-number burden and checkpoint-/angiogenesis-related anchor genes.

Candidate-gene analyses sharpened this contextual contrast. The IFNγ-associated side showed higher *IFNG*, *JAK1*, *STAT1*, *IRF1*, and *CD274* expression, whereas the myeloid-associated side showed higher *PDCD1LG2*, *SPP1*, *TREM2*, *APOE*, *TGFB1*, and *VEGFA* expression. Because several of these genes overlap with or are biologically proximal to the state-defining programs, these findings are described as characterization rather than independent validation. Mutation analyses showed relative *CTNNB1*-versus-*TP53* asymmetry across the operational labels, providing additional tumor-context information without implying causal determination of the immune axis (Fig 6B,C and Table S5 in S2 File).

Copy-number and methylation analyses provided weaker same-direction context for the myeloid-oriented end. Global absolute copy-number burden was modestly higher on the myeloid-associated side, selected gene-level copy-number values trended in the same direction, and methylation at selected checkpoint/*IFNG*-related loci was also higher. These signals were smaller and less consistent than the transcriptomic, single-cell, spatial, proteomic, and phosphoproteomic evidence and are therefore interpreted only as supportive molecular context (Fig 6D; Table S5 in S2 File).

## Discussion

This analysis supports a structured IFNγ-responsive-to-myeloid-suppressive immune continuum within immune-active HCC rather than two definitively discrete immune populations. Mixture models detected internal structure, but the unimodal kernel-density pattern, the absence of bootstrap evidence for multiple prominent density modes, and incomplete stability of the two-component assignment did not justify interpreting the fitted mixtures as naturally discrete biological populations. We therefore treat the continuous axis as primary and retain the IFNγ-responsive-associated and myeloid-suppressive-associated labels only as operational representations of opposing biological tendencies. This reframing is consistent with prior single-cell studies showing marked multicellular and intertumoral immune heterogeneity in HCC [6,7], while still allowing a compact transfer rule to summarize opposing ends of the biology.

The biological rationale for the axis is grounded in prior HCC immunology rather than discovered solely from the same defining genes. IFNγ signaling, antigen presentation, cytotoxic T-cell programs, and checkpoint activity are well-established features of immune-engaged HCC, whereas suppressive macrophage, stromal, TGFβ/VEGF, ECM, and hypoxia programs can constrain effective immunity [6,7,11,24–26]. Transcriptomic support from strict non-overlap gene sets was asymmetric. Strict non-overlap bulk analyses robustly supported antigen-processing and T-cell-effector programs at the IFNγ-oriented end, while the sample-aware single-cell analysis showed concordant strict non-overlap antigen-processing support; in contrast, strict non-overlap macrophage and fibroblast/ECM transcriptomic programs were weaker or nonsignificant. We therefore do not use the bulk or single-cell transcriptomic analyses alone to establish the myeloid-oriented ecology. Instead, stronger cross-modal support for this end derives from the paired PDC000198 and PDC000199 analyses: PDC000198 showed higher strict non-overlap macrophage, fibroblast/ECM, and hypoxia protein programs toward the myeloid-oriented end, whereas PDC000199 showed stronger SPP1-integrin-FAK, myeloid-receptor, fibroblast/ECM, and hypoxia-associated phosphorylation toward the same end. Together, these findings support a myeloid-stromal signaling ecology without relying on the original transcriptomic state-defining genes alone.

Independent HCC studies provide convergent external context for this interpretation. Single-cell and spatial analyses have localized SPP1 + macrophages to hypoxic tumor niches with active SPP1–CD44/integrin communication, while more recent spatial and functional work has demonstrated SPP1 + macrophage–ITGA5 + fibroblast crosstalk in HCC [27,28]. These studies do not validate the present axis directly, but they strengthen the biological plausibility of the recurrent macrophage–fibroblast–hypoxia/SPP1 signaling pattern observed across our proteomic and phosphoproteomic layers.

Importantly, the contribution of the present study is not the rediscovery of an IFNγ-high HCC phenotype or the assertion of a new binary immune taxonomy. Rather, it is the demonstration that heterogeneity within immune-active HCC can be represented as a transferable continuous coordinate whose opposing myeloid/stromal tendency is supported across molecular layers after explicitly separating reuse of defining genes from strict non-overlap and cross-modal evidence. This framework complements prior categorical HCC immune classifications [5,6] by preserving intermediate and mixed tumors and by providing a cross-platform deployment coordinate for a suppressive end that is less well captured by canonical inflamed signatures. The 10-gene panel should therefore be interpreted only as a deployment layer for this biological axis, not as a uniquely optimal feature set or a new clinical predictor. Its inclusion of established IFNγ-related genes such as *IFNG*, *CXCL9*, and *PRF1* makes recovery of the inflamed end expected rather than novel [24–26]. The lack of clear superiority over simpler immune signatures in the small treatment cohorts further limits claims of added predictive value. Instead, the recurrent macrophage, fibroblast/ECM, hypoxia, SPP1-integrin-FAK, and myeloid-receptor signals are best viewed as convergent molecular hypotheses for the myeloid-oriented ecology. Because the available public observational datasets do not provide perturbational, pharmacologic, or randomized evidence, targetability cannot be inferred; functional perturbation and pharmacologic studies are required to establish causality and therapeutic actionability.

The GSE206325 projection was designed as an exploratory biological bridge: fixed responder-associated transcriptional programs derived in treated HCC were scored in untreated HCC to ask whether the pre-existing immune axis showed directional resemblance to treatment-associated biology. Because the projection cohorts did not contain corresponding immunotherapy-response outcomes, this analysis cannot estimate response probability or predictive discrimination. More importantly, direct application of the finalized frozen transfer score to four public HCC immunotherapy cohorts produced heterogeneous and imprecise effects (pooled Hedges’ g = 0.10, 95% CI −0.42 to 0.61), providing insufficient evidence for clinical response prediction or incremental predictive value. Moreover, because the available HCC treatment cohorts lack matched non-ICI comparator arms, they cannot establish a biomarker-by-treatment interaction or distinguish treatment-specific predictive value from prognostic or other response-associated biology. Cross-cancer cohorts are therefore retained only as secondary contextual analyses. Prospective pretreatment HCC cohorts with harmonized treatment and response assessment, sufficient statistical power, and, where feasible, appropriate non-ICI or alternative-treatment comparator arms will be required to determine whether the axis has treatment-specific predictive utility rather than merely prognostic or other response-associated value.

Several limitations remain. To our knowledge, there is no universally accepted gold-standard label for the specific IFNγ-versus-myeloid ecologies studied here, and the operational labels depend on a predefined biological axis and operational cutoffs even though the underlying biology is continuous. All analyses were retrospective secondary analyses of public datasets, and no new functional experiments, prospectively collected tissues, or independent clinical trial samples were generated. In addition, the historical checkpoint-top-30% entry route used for compact-panel construction was selected during an exploratory TCGA screen that included OS information. Although survival is not used as validation in the revised study and the principal biological polarity was preserved across outcome-independent checkpoint thresholds and alternative immune-active entry definitions, this historical selection process may still have influenced the composition of the frozen deployment rule. Some cross-modal layers were incomplete for the compact panel: PDC000198 measured 6 of 10 proteins and PDC000199 only 3 of 10 phosphoproteins, so these datasets support pathway ecology rather than direct validation of the full 10-gene transfer score. The GSE206325 treatment-program derivation used sample-level pseudobulk profiles rather than independent patient-level observations and was therefore restricted to descriptive program construction; patient-balanced pretreatment validation will be needed to establish the robustness of these treatment-associated programs. GSE149614 likewise contains multiple anatomical specimens from some patients; sample-aware pseudobulk aggregation reduces cell-level pseudoreplication but does not make all anatomical-sample correlations patient-independent. Spatial quantification was available in only two analyzable sections, and the public HCC immunotherapy cohorts were small and heterogeneous in assay, treatment, timing, and endpoint definition. The spatial permutation analysis uses a simple count-preserving random-label null over fixed spot coordinates. This controls for endpoint abundance but does not preserve all higher-order spatial autocorrelation or tissue-architecture constraints; accordingly, the result is interpreted as reduced cross-end mixing relative to random label assignment, not as evidence of active biological repulsion or a complete mechanistic model of spatial segregation. Upstream mutation, copy-number, and methylation associations are contextual rather than causal. Accordingly, the study is positioned as a reproducible public-data framework for biological organization and positioning along a continuous immune axis, with hypothesis generation as its principal translational output, rather than as a finalized clinical assay.

## Conclusions

Immune-active hepatocellular carcinoma shows structured variation along an IFNγ-responsive-to-myeloid-suppressive continuum rather than two demonstrably discrete populations. A compact rank-based transfer score captured this axis across independent public HCC datasets. Non-overlapping transcriptomic analyses supported the IFNγ-oriented end, whereas spatial, proteomic, and phosphoproteomic analyses provided complementary evidence for a myeloid-stromal ecology involving macrophage, fibroblast/ECM, hypoxia, SPP1-integrin-FAK, and myeloid-receptor programs. Direct analyses of available public HCC immunotherapy cohorts were heterogeneous and did not establish a validated response predictor. The framework should therefore be viewed as a transferable representation for positioning tumors along a continuous immune axis and for generating testable biological hypotheses; prospective HCC cohorts and functional perturbation studies are required before clinical or therapeutic claims can be made.

## Supporting information

S1 File Supplementary Materials. This file contains supplementary methods and figure legends for discovery-route selection, continuous-axis sensitivity, alternative immune-active entry definitions, external transfer, single-cell and spatial immune-ecology mapping, proteomic/phosphoproteomic characterization, direct HCC treatment-cohort evaluation, and upstream molecular anchoring.
(DOCX)

S2 File Supplementary tables. This workbook contains the supplementary tables supporting the analyses reported in the manuscript.
(XLSX)

## Abbreviations

AIC: Akaike information criterion
AUC: area under the receiver operating characteristic curve
BIC: Bayesian information criterion
CI: confidence interval
ECM: extracellular matrix
FDR: false discovery rate
GEP: gene-expression profile
HCC: hepatocellular carcinoma
HR: hazard ratio
ICI: immune checkpoint inhibitor
ICGC: International Cancer Genome Consortium
IFNγ: interferon-gamma
OS: overall survival
PDC: Proteomic Data Commons
PFI: progression-free interval
PFS: progression-free survival
RECIST: Response Evaluation Criteria in Solid Tumors
RFS: recurrence-free survival
SD: standard deviation
TCGA: The Cancer Genome Atlas
