# Supplementary material for: Integrated multi-omic analysis characterizes an IFNγ-responsive-to-myeloid-suppressive immune continuum in immune-active hepatocellular carcinoma
Source: PLoS One. 2026 Oct 6;21(10):e0359327. doi: 10.1371/journal.pone.0359327 (PMC13641348; doi:10.1371/journal.pone.0359327)
Supplement: Supplementary file 1 [file pone.0359327.s001.docx]

**Supplementary Materials**

**Integrated multi-omic analysis characterizes an IFNγ-responsive-to-myeloid-suppressive immune continuum in immune-active hepatocellular carcinoma**

# **Table of Contents**

1. Supplementary Materials & Methods

S1. Cohort acquisition and preprocessing

S2. Immune-axis modules and scoring

S3. Immune-active entry, threshold sensitivity, and continuum analyses

S4. Compact classifier construction and external transfer quality control

S5. Single-cell projection and sample-aware cellular analyses

S6. Spatial transcriptomic reconstruction and ecology analyses

S7. Proteomic and phosphoproteomic cross-modal analyses

S8. Treatment-program mapping and direct HCC immunotherapy evaluation

S9. Upstream multi-omic anchoring analyses

S10. Statistical implementation and software versions

2. Supplementary Figures

S1 Fig. Outcome-independent sensitivity and strict non-overlap transcriptomic support.

S2 Fig. Supplementary clinical-context support for external classifier transfer.

S3 Fig. Supplementary support for single-cell communication and spatial immune-ecology mapping.

S4 Fig. Detailed support for direct HCC immunotherapy evaluation.

S5 Fig. Supplementary support for upstream molecular anchoring in TCGA-LIHC.

# **Supplementary Materials & Methods**

**S1. Cohort acquisition and preprocessing**

Public datasets were assembled with prespecified analytical roles. TCGA-LIHC served as the discovery and continuity backbone; GSE14520 and ICGC-LIRI-JP were used for frozen external HCC transfer. GSE149614 and GSE245908 provided sample-aware single-cell and spatial localization analyses, respectively, and PDC000198/PDC000199 provided paired proteomic and phosphoproteomic cross-modal support. GSE206325 was used only to derive treatment-associated responder programs. GSE140901, GSE215011, GSE279750, and GSE202069 were used for direct frozen evaluation in HCC immunotherapy cohorts. GSE285963 was used only for a supportive paired pre-/later-treatment analysis. GSE78220, GSE91061, and IMvigor210 were retained only as secondary cross-cancer treatment context. Dataset-level provenance, sample-selection rules, endpoint or contrast definitions, and preprocessing scope are summarized in Supplementary Table S1E.

**S2. Immune-axis modules and scoring**

Curated checkpoint, inflamed, myeloid, antigen-presentation, and T-cell-support modules were specified to operationalize literature-motivated immune poles. The inflamed and myeloid modules and the direction of the continuous inflamed-minus-myeloid axis were defined before survival or immunotherapy-response analyses and were not selected or optimized using clinical outcomes. Gene-wise z-scores were calculated across samples and module scores were obtained as the mean standardized expression of available component genes. The continuous inflamed-minus-myeloid score was treated as the primary biological phenotype in the analytical framework.

**S3. Immune-active entry, threshold sensitivity, and continuum analyses**

The checkpoint top-30% subset is retained as the historical discovery subset used to construct the frozen compact classifier. In the original exploratory workflow, checkpoint entry windows spanning the top 20%, 25%, 30%, and 35% and several partition rules were compared, and the historical route screen included survival information. Accordingly, we do not interpret the top-30% threshold as uniquely optimal and do not use TCGA survival performance as validation of the immune framework. Outcome-independent sensitivity analyses were performed across checkpoint top-20%, top-25%, top-30%, and top-35% entry windows. Within each subset, the continuous axis was recalculated from the same fixed modules and median-based labels were used only for descriptive polarity comparisons. Sample-set overlap, operational-label concordance, and Hedges' g for checkpoint, inflamed, myeloid, antigen-presentation, and T-cell-support modules were compared across thresholds. We additionally evaluated seven alternative top-30% immune-active definitions based on an immune composite, inflamed score, cytolytic activity, T-cell support, CD8 expression, immune infiltration, and a T-cell-inflamed gene-expression profile. Finally, one-, two-, and three-component Gaussian mixture models, kernel-density mode counting, Ashman’s D, silhouette coefficients, and 1,000 nonparametric bootstrap resamples generated with the fixed random seed specified in the deposited code were used to assess distributional structure and assignment stability. For the continuity diagnostics, axis values were standardized to zero mean and unit population variance before Gaussian-mixture fitting. Mixtures were fitted with scikit-learn GaussianMixture using n_init = 20 and random_state = 20. Full-sample initialization robustness was additionally assessed across random_state values 0–99, each with n_init = 20. Kernel density was estimated using SciPy gaussian_kde with its default bandwidth on a 1,024-point grid extending 0.5 standard deviations beyond the observed minimum and maximum; peaks were counted as prominent when their prominence was at least 10% of the maximum estimated density. For bootstrap assignment stability, each two-component bootstrap fit was projected back to the original observations on the bootstrap-standardized scale and compared with the full-sample two-component assignment using the adjusted Rand index. Mixture-component count was treated only as a distributional diagnostic rather than as a biological class count, and the historical median split was used solely to generate operational end-group labels for descriptive comparisons and compact-panel construction. Numerical continuity and modality findings are reported in the main Results, Fig 2B, S1 Fig C, and Supplementary Table S1A.

**S4. Compact classifier construction and external transfer quality control**

A compact panel was derived from the union of genes in the prespecified checkpoint, inflamed, myeloid, antigen-presentation, and T-cell-support modules using the previously assigned operational TCGA end-group labels. Candidate genes were ranked by the absolute mean standardized-expression difference between the IFNγ-responsive-associated and myeloid-suppressive-associated end groups, with representation retained from both directional arms. Feature ranking was performed once in the full historical TCGA discovery subset before cross-validation and was not repeated within individual training folds. Accordingly, the fivefold cross-validation metric was used only as a historical panel-size screening heuristic and is not interpreted as an unbiased estimate of out-of-sample performance. Candidate panel sizes of 10–16 genes were evaluated by fivefold stratified cross-validation using a nearest-centroid classifier. For each candidate size, balanced accuracy and class-specific recalls were calculated, and the historical optimization score was defined as balanced accuracy − 0.10 × |recallIFNγ − recallmyeloid|. The historical workflow retained the 10-gene candidate according to this panel-size screening criterion. The selected panel comprised five genes retained from the historical IFNγ-associated selection direction (*CXCL9*, *B2M*, *IFNG*, *CTLA4*, and *PRF1*) and five genes retained from the historical myeloid-associated selection direction (*VEGFA*, *TREM2*, *TGFB1*, *APOE*, and *SPP1*). These historical selection directions refer to the standardized-expression screening stage; the final frozen template-contrast weights were recalculated after stability-enriched within-sample rank recentering and therefore need not preserve the same sign for every individual gene. Panels smaller than 10 genes were not evaluated, so this historical screen does not establish 10 genes as a minimum sufficient or globally optimal panel size.

After the panel had been selected, stability-enriched re-centering and final deployment-rule construction were performed without reopening gene selection. The frozen external workflow used within-sample panel ranks, template-contrast weights, class-specific uncertainty thresholds, intermediate-category handling, and minimum panel-overlap rules. The template-contrast output is a continuous signed transfer score aligned with the IFNγ-to-myeloid direction. The fixed thresholds are applied only when categorical operational labels are needed; samples falling between the two class-specific thresholds remain intermediate. Accordingly, the continuous transfer score and the thresholded labels are two representations of the same frozen deployment rule rather than competing biological definitions. GSE14520 and ICGC-LIRI-JP were not used to reselect genes or optimize thresholds. Sensitivity was assessed at the panel-size level (Fig 3A) and for the missing-gene pattern encountered in GSE140901: omission of *B2M* in TCGA yielded Pearson r = 0.994 and Spearman rho = 0.989 between the complete and *B2M*-omitted continuous scores, 92.5% sign concordance, and 84.1% three-class concordance. These analyses support portability and robustness of the compact deployment layer but do not establish unique feature optimality or clinical predictive validity. Selection and sensitivity provenance is summarized in Supplementary Table S2D.

**S5. Single-cell projection and sample-aware cellular analyses**

GSE149614 was reanalyzed with exact cell-identifier matching to metadata. The frozen 10-gene template-contrast score was calculated primarily from sample-level and sample-by-cell-type pseudobulk profiles, while per-cell scores were retained for descriptive localization. Because some patients contributed multiple anatomical specimens, sample-level pseudobulk aggregation reduced cell-level pseudoreplication but did not render all anatomical-sample analyses patient-independent. Accordingly, correlations across anatomical samples were treated as descriptive sample-level associations, whereas paired tumor-versus-non-tumor comparisons were evaluated within patient pairs. The strict non-overlap programs excluded every compact-panel gene and all genes used in the original checkpoint, inflamed, and myeloid defining modules. For the sample-level GSE149614 strict non-overlap analysis, the analysis set comprised the 13 tumor-related anatomical specimens and excluded the eight non-tumor liver specimens. The five program scores (antigen-processing, T-cell-effector, macrophage, fibroblast/ECM, and hypoxia) were evaluated separately against the frozen 10-gene template-contrast score by two-sided Spearman correlation. Raw P values from these five correlations constituted one prespecified correction family and were adjusted together using the Benjamini–Hochberg procedure. The antigen-processing program yielded rho = 0.758, raw P = 0.002666, and FDR = 0.01333. CellChat and focused NicheNet analyses were retained as hypothesis-generating communication-pathway characterization rather than independent validation or functional evidence.

**S6. Spatial transcriptomic reconstruction and ecology analyses**

The GSE245908 spatial dataset was reconstructed into sample-wise objects and projected using the continuous directional framework. Spot-level axis scores, reference-bridge correlations, neighborhood organization, nearest IFNγ-high-to-myeloid-high distance, and Ripley-based spatial summaries were evaluated in the two analyzable sections. To define the primary local-neighborhood radius independently of the IFNγ-high/myeloid-high spot labels or the observed nearest-distance distribution, direct Visium lattice-neighbor spot pairs were identified from array coordinates using the three forward hexagonal-neighbor offsets (0,+2), (+1,-1), and (+1,+1). Full-resolution center-to-center distances were then calculated from the source tissue-position files. The largest direct-neighbor distance across CHC20 and CHC23 was 221.777 pixels; its ceiling, 222 pixels, was therefore used as the primary radius corresponding to one direct Visium lattice-neighbor center-to-center step. This value is distinct from the spot diameter (~143 full-resolution pixels in these sections). Because cell-fraction correlations with the continuous axis were weak and section-dependent, the spatial results are interpreted as local proximity and coexistence of IFNγ-high and myeloid-high programs rather than universal one-to-one cellular co-localization. Representative annotations in the main Fig 4D were selected algorithmically from the same neighborhood analysis for visualization only; statistical interpretation was based on the complete section-level proximity summaries.

To distinguish frequent absolute proximity from preferential spatial mixing, we additionally performed 10,000 count-preserving within-section label permutations using fixed seed 20260809. The frozen IFNγ-high, myeloid-high, and intermediate labels were randomly reassigned over the fixed spot coordinates while preserving category counts, and the fraction of IFNγ-high spots with at least one myeloid-high spot within 222 pixels was recalculated. Empirical tail probabilities used a +1 correction, and two-sided empirical P values were calculated as twice the smaller tail probability, capped at 1. This null controls for category abundance but does not preserve higher-order spatial autocorrelation or tissue architecture and is therefore interpreted only as an abundance-matched random-label reference.

**S7. Proteomic and phosphoproteomic cross-modal analyses**

PDC000198 and PDC000199 were analyzed as paired cross-modal molecular layers from 165 HCC and 165 adjacent non-tumoral samples. PDC000198 contained 6 of the 10 compact-panel proteins and was therefore used for an exploratory partial-panel protein-axis projection and strict non-overlap protein programs rather than exact classifier validation. For PDC000199, RefSeq protein accessions were mapped to human gene symbols using NCBI gene-product records; 48,992 of 49,683 phosphosites (98.6%) were mapped, yielding 7,028 gene-level phosphorylation profiles after median aggregation of multiple phosphosites per gene. Because only 3 of the 10 compact-panel genes were represented at the phosphoprotein level, PDC000199 was analyzed using prespecified signaling programs, including SPP1-integrin-FAK, myeloid-receptor, fibroblast/ECM, hypoxia, IFNG-JAK-STAT, VEGF-PI3K-AKT-MAPK, MIF-CD74-CXCR4, and ECM-FAK-SRC. These layers are interpreted as cross-modal biological support, not as direct compact-classifier validation.

**S8. Treatment-program mapping and direct HCC immunotherapy evaluation**

GSE206325 was used only to derive an exploratory treatment-associated transcriptional bridge. The processed SingleCellExperiment object and the source Liver_Treated_patients sample annotation were used to link cells to biological sample identifiers and source-study response labels. Cells with mappable sample and response labels were summed within sample to create pseudobulk counts, normalized by library size to counts per million, and log1p transformed. The retained derivation set contained 92 responder-labeled and 176 non-responder-labeled pseudobulk profiles; these are profile counts rather than independent patient counts. For each gene, the responder-associated effect was the responder-group mean minus the non-responder-group mean. The top 50 positive-effect genes and top 50 negative-effect genes were frozen as responder-up and responder-down programs, respectively. In untreated TCGA-LIHC and the locked GSE14520 expression matrix, program scores were calculated as the mean within-sample percentile rank of available genes, with responder axis defined as responder-up minus responder-down. Operational-group summaries used the previously assigned operational labels and were descriptive only; no treatment-response outcome was available in these projection cohorts.

The previously frozen 10-gene template-contrast rule was then applied without retraining or outcome-based retuning to GSE140901, GSE215011, GSE279750, and GSE202069. Continuous-score associations were summarized using cohort-specific AUCs, Hedges’ g, Mann–Whitney tests, odds ratios per standard-deviation increase where estimable, bootstrap confidence intervals, and random-effects pooling. The frozen 10-gene template-contrast score and seven comparator scores were evaluated in the same four HCC cohorts using the same cohort-specific endpoints. Comparator scores used the original prespecified discovery-module definitions; AUC confidence intervals were obtained from 5,000 nonparametric bootstrap resamples, discarding single-class resamples, and AUCs were not pooled. For GSE202069, gene-wise comparator standardization was performed in the full source expression matrix before restriction to the 17 anti-PD-1-treated patients. These direct HCC analyses, rather than the untreated projection, define the present treatment-response claim boundary.

For GSE202069, treatment eligibility was defined directly from the source clinical field PD1.therapy = Yes, yielding 17 treated patients with available response annotation (8 responders and 9 non-responders). Sample-name prefixes were not used to define treatment exposure, and identical numeric suffixes were not treated as global patient identifiers across different prefix groups. Exploratory survival analyses used the frozen 10-gene template-contrast score standardized within these 17 treated patients as the sole covariate in separate Cox proportional-hazards models for PFS and OS. We report the log-hazard coefficient (β), its standard error, Wald z statistic, hazard ratio per one-standard-deviation increase, 95% confidence interval, and two-sided Wald P value. These survival analyses were descriptive, were not multiplicity-adjusted, and were not used to support prognostic, predictive, or treatment-specific claims. For the supportive tissue-pair analyses, pairing was restricted to the source-defined CT–CP control series (n = 13 pairs) and ST–SP surgery series (n = 12 pairs); shared numeric suffixes across unrelated prefixes were not used to infer patient identity.

GSE285963 was used only for a supportive paired treatment-timepoint analysis. Eleven patients had a pretreatment sample and one later sample available for the analyzed expression matrix; the later sample was labeled post-treatment for 10 patients and on-treatment for one patient. Within-patient later-minus-pre changes in the frozen 10-gene template-contrast score were summarized by the median and paired Wilcoxon test. GSE285963 was not included in the four-HCC response meta-analysis.

**S9. Upstream multi-omic anchoring analyses**

Upstream analyses were restricted to prespecified expression-space axes, candidate-gene contrasts, mutation asymmetry, copy-number support, and methylation support in TCGA-LIHC. These analyses provide molecular context for variation along the continuous immune axis and are not interpreted as causal determinants of discrete biological classes.

**S10. Statistical implementation and software versions**

Analyses were performed using Python- and R-based workflows. The accompanying repository provides a portable Conda environment specification (environment.yml), a Python package snapshot (software_versions_python.txt), and an R runtime/session snapshot (software_versions_R.txt). The archived R snapshot records R 4.6.0 but does not establish exact versions of all analytical R packages because those packages were not loaded in that session, and the archived Python package snapshot does not contain an independent interpreter-version record. The portable dependencies required to recreate the analysis environment are therefore specified in environment.yml, whereas the archived version files are retained as provenance snapshots. Exact interpreter or package versions are reported only where directly documented by the archived records.

Statistical analyses used two-sided testing, Benjamini-Hochberg false-discovery-rate correction where multiple prespecified features were tested, Hedges' g for standardized group contrasts, Spearman correlation for continuous associations, paired Wilcoxon tests for paired tissue analyses, and random-effects meta-analysis for small heterogeneous treatment cohorts. For the four-HCC treatment synthesis, DerSimonian–Laird random-effects pooling was used for the primary Hedges’ g estimate. Because only four direct HCC treatment cohorts were available, a sensitivity analysis estimated τ² by restricted maximum likelihood and applied the Hartung–Knapp adjustment to the pooled confidence interval. This small-k sensitivity analysis was used to assess robustness of the inferential conclusion rather than to select a more favorable estimate. Continuity was evaluated with Gaussian-mixture BIC, Ashman's D, kernel-density mode counting, silhouette coefficients, and bootstrap stability. Survival results from the historical TCGA route screen are not used as validation of the immune framework.

# **Supplementary Figures**

**S1 Fig. Outcome-independent sensitivity and strict non-overlap transcriptomic support.**

(A) Sensitivity of sample selection and operational end-group labeling across checkpoint-high entry windows spanning the top 20%, 25%, 30%, and 35% of the checkpoint-score distribution. The Jaccard index compares each selected sample set with the historical checkpoint-top-30% subset, and label concordance is calculated among overlapping samples after re-estimating the median continuous-axis cutoff within each window. (B) Pairwise Jaccard overlap among eight alternative top-30% immune-active definitions, demonstrating that different entry measures select only partially overlapping tumors despite preservation of the same overall biological polarity. (C) Bootstrap stability of the two-component mixture representation. Each point shows Ashman's D for the two-component fit versus the adjusted Rand index relative to the original two-component assignment; the vertical dashed line marks Ashman's D = 2. None of the 1,000 fixed-seed bootstrap resamples contained two or more prominent kernel-density modes, and the median bootstrap adjusted Rand index for the two-component assignment was 0.504. (D) Threshold-specific continuous associations between the inflamed-minus-myeloid axis and strict non-overlap antigen-processing, T-cell-effector, macrophage, fibroblast/ECM, and hypoxia programs across checkpoint top-20%, top-25%, top-30%, and top-35% entry windows. All compact-panel and original state-defining genes were excluded from these programs.

**S2 Fig. Supplementary clinical-context support for external classifier transfer**

(A) GSE14520 overall-survival analysis using the frozen thresholded non-intermediate operational labels. (B) GSE14520 recurrence-free survival analysis using the same thresholded operational labels. (C) Donor-collapsed LIRI-JP overall-survival analysis showing supportive but non-significant external directionality.

**S3 Fig. Supplementary support for single-cell communication and spatial immune-ecology mapping.**

(A) Additional sample-level single-cell compartment summaries in GSE149614. (B) Additional CellChat sender-receiver summaries and target-axis interaction tables supporting the suppressive-remodeling communication program; these analyses are interpreted as hypothesis-generating communication-pathway characterization rather than independent validation or functional evidence. (C) GSE245908 CHC20 spatial IFNγ, myeloid, and continuous inflamed-minus-myeloid spatial-ecology maps. (D) GSE245908 CHC23 spatial IFNγ, myeloid, and continuous inflamed-minus-myeloid spatial-ecology maps. Quantitative spatial correlations, observed cross-end contact fractions, abundance-matched random-label permutation comparisons, and IFNγ-high-to-myeloid-high nearest-distance statistics are summarized in Fig 4D and the main text.

**S4 Fig. Detailed support for direct HCC immunotherapy evaluation.**

(A) Sample-level frozen 10-gene template-contrast score distributions in GSE140901, GSE215011, GSE279750, and GSE202069, shown separately for responders and non-responders using each cohort’s prespecified binary treatment endpoint; GSE140901 used clinical benefit as the endpoint. (B) Cohort-specific odds ratios per one-standard-deviation increase in the frozen 10-gene template-contrast score and the random-effects four-HCC pooled estimate. (C) Leave-one-cohort-out sensitivity of the four-HCC pooled Hedges' g. (D) GSE140901 *B2M*-dropout sensitivity, showing Pearson and Spearman correlations, continuous-score sign concordance, and three-class deployment concordance between the complete and *B2M*-omitted TCGA scores. (E) Cohort-specific exploratory benchmark heatmap for the frozen 10-gene template-contrast score and seven comparator immune signatures across GSE140901, GSE215011, GSE279750, and GSE202069, summarized by Hedges’ g using each cohort’s prespecified response endpoint. (F) Supportive paired changes in the frozen 10-gene template-contrast score in GSE285963 and in the control and surgery comparisons from GSE202069. Panels E-F are exploratory and are not interpreted as validated response prediction.

**S5 Fig. Supplementary support for upstream molecular anchoring in TCGA-LIHC.**

(A) Ranking of prespecified upstream anchors by directional difference between operational IFNγ-responsive-associated and myeloid-suppressive-associated tumors. (B) Detailed candidate-gene differences across inflammatory, suppressive-remodeling, and tumor-associated markers. (C) Upstream anchor-panel matrix across prespecified features. (D) Evidence map linking inflammatory, suppressive-remodeling, and tumor-associated chains to their supporting molecular layers. (E) Coverage summary of prespecified upstream anchors across the analyzed molecular layers. (F) Compact summary of the strongest positive and negative upstream anchors.
