# Supplementary material for: Integrated multi-omic analysis characterizes an IFNγ-responsive-to-myeloid-suppressive immune continuum in immune-active hepatocellular carcinoma
Source: PLoS One. 2026 Oct 6;21(10):e0359327. doi: 10.1371/journal.pone.0359327 (PMC13641348; doi:10.1371/journal.pone.0359327)
Supplement: Supplementary file 2 [file pone.0359327.s003.docx]

Lin et al propose a computational framework to stratify immune-active HCC into two immune states, I**FNγ-responsive** and **myeloid-suppressive** state. A 10-gene rank-based classifier was developed and transferred to independent cohorts, with additional support from single-cell RNA-seq, spatial transcriptomics, proteomics, and public immunotherapy datasets. The study is technically comprehensive and integrates multiple public datasets under a frozen analytical framework. However, the biological novelty, rationale for the classification strategy, and translational significance remain insufficiently established. In its current form, the manuscript appears to provide a computational refinement of an already recognized IFNγ–myeloid immune axis rather than a substantial conceptual advance, and several methodological issues require clarification.

**Major Comments**

1. Biological rationale for the two-state hypothesis is insufficient. The authors assumed that immune-active HCC comprises two discrete immune states but the rationale for this hypothesis is not clearly established. If this model is based on previous literature, the relevant studies should be discussed.

2. The authors defined immune-active tumors using the top 30% of checkpoint expression. The rationale for selecting checkpoint expression as the entry criterion over other established measures (e.g., immune infiltration, cytolytic activity, T-cell activation, immune scores) is unclear.

Furthermore, the data appear to support a continuous immune axis rather than two clearly separated biological populations, as acknowledged by the authors. Given that most tumors cluster within a similar region (Fig. 2B, most samples fell in the lower left region) of the inflamed-versus-myeloid space, the biological justification for imposing a binary classification is not convincing.

3. The discovery framework is partially circular. The proposed immune states were defined using inflamed and myeloid immune programs, yet subsequent validations showed that IFNγ-responsive state exhibited higher IFNγ signaling, checkpoint activity, antigen presentation, and T-cell-support signatures, while the other showed stronger myeloid programs. These downstream analyses are confirmatory rather than independent validation. Greater emphasis should instead be placed on orthogonal evidence to demonstrate that the proposed states capture novel biology beyond the discovery framework.

4. The biological and translational significance of the proposed classification remains unclear. While subdividing immune-active HCC is potentially valuable, the manuscript does not demonstrate how this classification advances current understanding or improves clinical decision-making. It remains unclear whether these immune states better predict immunotherapy response, identify distinct therapeutic vulnerabilities, or provide information beyond existing immune classification systems. The multi-omic analyses are largely descriptive and do not substantially extend the biological findings from the discovery cohort. Applying the finalized 10-gene classifier directly to HCC immunotherapy cohorts and evaluating its predictive performance would substantially strengthen the translational impact.

5. The classifier largely recapitulates existing immune-active signatures. Several genes included in the 10-gene classifier (IFNG, CXCL9, PRF1) are already widely used to define inflamed or immune-active tumors. Consequently, identifying an IFNγ-responsive subgroup is expected and provides limited additional biological insight. The manuscript would be strengthened by focusing more deeply on the biological characteristics and therapeutic relevance of the myeloid-suppressive subgroup, which represents the more novel aspect of the study.

6. The manuscript states that projection into untreated HCC cohorts demonstrates higher responder-up signals and favorable responder-axis shifts in the IFNγ-responsive state. Can the author explain more in the manuscript as to how they arrived at this conclusion?

Further, the distinction between transcriptional similarity to responder programs and validated prediction of immunotherapy benefit should be clarified throughout the manuscript.

**Minor Comments**

1. The manuscript states that a biologically curated 10-gene panel was selected but does not adequately explain why these specific genes or why exactly ten genes were chosen. I could not fully extrapolate the information regarding feature selection, robustness, or sensitivity analyses from this manuscript.

2. Throughout the Results section, conclusions are often difficult to associate with the corresponding figures. More precise figure citations would improve readability.

3. Figure 4C alone does not adequately support the conclusions. Supporting analyses currently presented in the Supplementary Figures should be incorporated into the main figures, and regions demonstrating co-localization of IFNγ-like and myeloid-like states should be more clearly annotated.

4. Manuscript lacks overarching roadmap. It would be helpful to sufficiently map out the logic of the paper when describing Fig.1 to orientate the readers.
