# Supplementary material for: Integrated multi-omic analysis characterizes an IFNγ-responsive-to-myeloid-suppressive immune continuum in immune-active hepatocellular carcinoma
Source: PLoS One. 2026 Oct 6;21(10):e0359327. doi: 10.1371/journal.pone.0359327 (PMC13641348; doi:10.1371/journal.pone.0359327)
Supplement: Peer review history

# Peer review: Decision Letter 0

**DOI:** 10.1371/journal.pone.0359327.r001

**Authors:** Kangkang Ji

6 Aug 2026

Dear Dr. Xiao,

Thank you for submitting your manuscript to PLOS ONE. After careful consideration, we feel that it has merit but does not fully meet PLOS ONE’s publication criteria as it currently stands. Therefore, we invite you to submit a revised version of the manuscript that addresses the points raised during the review process.

Please submit your revised manuscript by Oct 05 2026 11:59PM. If you will need more time than this to complete your revisions, please reply to this message or contact the journal office at plosone@plos.org. When you're ready to submit your revision, log on to https://www.editorialmanager.com/pone/ and select the 'Submissions Needing Revision' folder to locate your manuscript file.

- A letter that responds to each point raised by the academic editor and reviewer(s). You should upload this letter as a separate file labeled 'Response to Reviewers'.
- A marked-up copy of your manuscript that highlights changes made to the original version. You should upload this as a separate file labeled 'Revised Manuscript with Track Changes'.
- An unmarked version of your revised paper without tracked changes. You should upload this as a separate file labeled 'Manuscript'.

If you would like to make changes to your financial disclosure, please include your updated statement in your cover letter. Guidelines for resubmitting your figure files are available below the reviewer comments at the end of this letter.

If applicable, we recommend that you deposit your laboratory protocols in protocols.io to enhance the reproducibility of your results. Protocols.io assigns your protocol its own identifier (DOI) so that it can be cited independently in the future. For instructions see: https://journals.plos.org/plosone/s/submission-guidelines#loc-laboratory-protocols. Additionally, PLOS ONE offers an option for publishing peer-reviewed Lab Protocol articles, which describe protocols hosted on protocols.io. Read more information on sharing protocols at https://plos.org/protocols?utm_medium=editorial-email&utm_source=authorletters&utm_campaign=protocols.

As the corresponding author, your ORCID iD is verified in the submission system and will appear in the published article. PLOS supports the use of ORCID, and we encourage all coauthors to register for an ORCID iD and use it as well. Please encourage your coauthors to verify their ORCID iD within the submission system before final acceptance, as unverified ORCID iDs will not appear in the published article. *Only*  the individual author can complete the verification step; PLOS staff *cannot*  verify ORCID iDs on behalf of authors.

We look forward to receiving your revised manuscript.

Kind regards,

Kangkang Ji, Ph.D., M.D.

Academic Editor

PLOS One

Journal Requirements:

When submitting your revision, we need you to address these additional requirements.

1. Please ensure that your manuscript meets PLOS ONE's style requirements, including those for file naming. The PLOS ONE style templates can be found at https://journals.plos.org/plosone/s/file?id=wjVg/PLOSOne_formatting_sample_main_body.pdf and https://journals.plos.org/plosone/s/file?id=ba62/PLOSOne_formatting_sample_title_authors_affiliations.pdf

2. Please note that PLOS One has specific guidelines on code sharing for submissions in which author-generated code underpins the findings in the manuscript. In these cases, all author-generated code must be made available without restrictions upon publication of the work. Please review our guidelines at https://journals.plos.org/plosone/s/materials-and-software-sharing#loc-sharing-code and ensure that your code is shared in a way that follows best practice and facilitates reproducibility and reuse.

3. Thank you for stating in your Funding Statement:

[This work was supported by the Natural Science Foundation of Fujian Province (grant numbers 2023J011296 and 2023J011295 to X.C.) and the Joint Funds for the Innovation of Science and Technology, Fujian Province (grant number 2021Y9218 to X.C.). The funder website is https://kjt.fujian.gov.cn/. The funders had no role in study design, data collection and analysis, decision to publish, or preparation of the manuscript.].

Please provide an amended statement that declares *all* the funding or sources of support (whether external or internal to your organization) received during this study, as detailed online in our guide for authors at http://journals.plos.org/plosone/s/submit-now.  Please also include the statement “There was no additional external funding received for this study.” in your updated Funding Statement.

Please include your amended Funding Statement within your cover letter. We will change the online submission form on your behalf.

If the reviewer comments include a recommendation to cite specific previously published works, please review and evaluate these publications to determine whether they are relevant and should be cited. There is no requirement to cite these works unless the editor has indicated otherwise.

[Note: HTML markup is below. Please do not edit.]

Reviewers' comments:

Reviewer's Responses to Questions

**Comments to the Author**

1. Is the manuscript technically sound, and do the data support the conclusions?

Reviewer #1: Partly

Reviewer #2: No

Reviewer #3: Yes

**********

2. Has the statistical analysis been performed appropriately and rigorously?

Reviewer #1: Yes

Reviewer #2: No

Reviewer #3: Yes

**********

3. Have the authors made all data underlying the findings in their manuscript fully available?

The PLOS Data policy

Reviewer #1: Yes

Reviewer #2: Yes

Reviewer #3: Yes

**********

4. Is the manuscript presented in an intelligible fashion and written in standard English?

Reviewer #1: Yes

Reviewer #2: Yes

Reviewer #3: Yes

**********

Reviewer #1: Lin et al propose a computational framework to stratify immune-active HCC into two immune states, IFNγ-responsive and myeloid-suppressive state. A 10-gene rank-based classifier was developed and transferred to independent cohorts, with additional support from single-cell RNA-seq, spatial transcriptomics, proteomics, and public immunotherapy datasets. The study is technically comprehensive and integrates multiple public datasets under a frozen analytical framework. However, the biological novelty, rationale for the classification strategy, and translational significance remain insufficiently established. In its current form, the manuscript appears to provide a computational refinement of an already recognized IFNγ–myeloid immune axis rather than a substantial conceptual advance, and several methodological issues require clarification.

Major Comments

1. Biological rationale for the two-state hypothesis is insufficient. The authors assumed that immune-active HCC comprises two discrete immune states but the rationale for this hypothesis is not clearly established. If this model is based on previous literature, the relevant studies should be discussed.

2. The authors defined immune-active tumors using the top 30% of checkpoint expression. The rationale for selecting checkpoint expression as the entry criterion over other established measures (e.g., immune infiltration, cytolytic activity, T-cell activation, immune scores) is unclear.

Furthermore, the data appear to support a continuous immune axis rather than two clearly separated biological populations, as acknowledged by the authors. Given that most tumors cluster within a similar region (Fig. 2B, most samples fell in the lower left region) of the inflamed-versus-myeloid space, the biological justification for imposing a binary classification is not convincing.

3. The discovery framework is partially circular. The proposed immune states were defined using inflamed and myeloid immune programs, yet subsequent validations showed that IFNγ-responsive state exhibited higher IFNγ signaling, checkpoint activity, antigen presentation, and T-cell-support signatures, while the other showed stronger myeloid programs. These downstream analyses are confirmatory rather than independent validation. Greater emphasis should instead be placed on orthogonal evidence to demonstrate that the proposed states capture novel biology beyond the discovery framework.

4. The biological and translational significance of the proposed classification remains unclear. While subdividing immune-active HCC is potentially valuable, the manuscript does not demonstrate how this classification advances current understanding or improves clinical decision-making. It remains unclear whether these immune states better predict immunotherapy response, identify distinct therapeutic vulnerabilities, or provide information beyond existing immune classification systems. The multi-omic analyses are largely descriptive and do not substantially extend the biological findings from the discovery cohort. Applying the finalized 10-gene classifier directly to HCC immunotherapy cohorts and evaluating its predictive performance would substantially strengthen the translational impact.

5. The classifier largely recapitulates existing immune-active signatures. Several genes included in the 10-gene classifier (IFNG, CXCL9, PRF1) are already widely used to define inflamed or immune-active tumors. Consequently, identifying an IFNγ-responsive subgroup is expected and provides limited additional biological insight. The manuscript would be strengthened by focusing more deeply on the biological characteristics and therapeutic relevance of the myeloid-suppressive subgroup, which represents the more novel aspect of the study.

6. The manuscript states that projection into untreated HCC cohorts demonstrates higher responder-up signals and favorable responder-axis shifts in the IFNγ-responsive state. Can the author explain more in the manuscript as to how they arrived at this conclusion?

Further, the distinction between transcriptional similarity to responder programs and validated prediction of immunotherapy benefit should be clarified throughout the manuscript.

Minor Comments

1. The manuscript states that a biologically curated 10-gene panel was selected but does not adequately explain why these specific genes or why exactly ten genes were chosen. I could not fully extrapolate the information regarding feature selection, robustness, or sensitivity analyses from this manuscript.

2. Throughout the Results section, conclusions are often difficult to associate with the corresponding figures. More precise figure citations would improve readability.

3. Figure 4C alone does not adequately support the conclusions. Supporting analyses currently presented in the Supplementary Figures should be incorporated into the main figures, and regions demonstrating co-localization of IFNγ-like and myeloid-like states should be more clearly annotated.

4. Manuscript lacks overarching roadmap. It would be helpful to sufficiently map out the logic of the paper when describing Fig.1 to orientate the readers.

Reviewer #2: I read with great interest the research article by Chen and colleagues. Overall, it represents an in-depth exploration of hepatocellular carcinoma. However, in terms of study design, writing quality, and ethical considerations, the current version cannot be published in PLOS ONE.

1.What is the gold standard of this study? The authors appear to have relied solely on assumptions, with no apparent gold standard. There is no wet-lab validation; the findings are derived only from database analysis.

2.Some of the content currently in the Results section should be placed in the Methods section. A substantial portion of the Results is written in a style more appropriate for the Methods. The authors appear to have an unclear understanding of the distinction between Methods and Results.

3.In Figure 4C, human tissue sections are presented. Although this is only one component of the validation, it still involves liver tissue sections and requires ethical approval. This issue cannot be overlooked!

4.The foundation of this study is based on a binary classification approach. However, the subsequent interpretation employs "frozen state-axis scores". This appears to be contradictory.

5.There is almost no clinical validation, and the findings do not fully or conclusively support the authors' conclusions.

Overall, substantial revision—or even rewriting—is still required in order to meet SCI publication standards.

Reviewer #3: This manuscript presents a public-data integration framework for characterizing immune heterogeneity in HCC. The integration of bulk, single-cell, spatial, proteomic, and treatment-associated datasets is potentially valuable and It’s promising. The manuscript need Minor revision.

The current stratification does not sufficiently demonstrate two naturally distinct immune states. Unless functional validation is added, more neutral terms such as the following would be better: IFNγ-responsive-associated state, Myeloid- suppressive-associasted state.

**********

what does this mean?). If published, this will include your full peer review and any attached files.

If you choose “no”, your identity will remain anonymous but your review may still be made public.

**Do you want your identity to be public for this peer review?**  For information about this choice, including consent withdrawal, please see our Privacy Policy

Reviewer #1: No

Reviewer #2: No

Reviewer #3: **Yes:**  ZONGYI WANG

**********

[NOTE: If reviewer comments were submitted as an attachment file, they will be attached to this email and accessible via the submission site. Please log into your account, locate the manuscript record, and check for the action link "View Attachments". If this link does not appear, there are no attachment files.]

To ensure your figures meet our technical requirements, please review our figure guidelines: https://journals.plos.org/plosone/s/figures

You may also use PLOS’s free figure tool, NAAS, to help you prepare publication quality figures: https://journals.plos.org/plosone/s/figures#loc-tools-for-figure-preparation.

NAAS will assess whether your figures meet our technical requirements by comparing each figure against our figure specifications.

Attachment Submitted filename: PONE-D-26-24025_reviewer comments.docx

# Author comment: Author response to Decision Letter 1

**DOI:** 10.1371/journal.pone.0359327.r002

12 Aug 2026

Response to the Editor

Dear Editor Kangkang Ji,

We sincerely thank you for the opportunity to revise manuscript PONE-D-26-24025 and for the clear guidance regarding scientific rigor, code availability, funding disclosure, and the reviewers' concerns. We have undertaken a comprehensive revision of the title, Abstract, Introduction, Materials and Methods, Results, Discussion, Conclusions, ethics statement, data/code availability statements, figure legends, and Supporting Information.

The central conceptual revision is that the manuscript no longer presents two naturally discrete immune populations. The continuous IFNγ-responsive-to-myeloid-suppressive axis is now the primary biological model, while the two labels are explicitly defined as operational, axis-associated representations of opposing tendencies. Formal modality analysis, alternative immune-active entry definitions, and strict non-overlap sensitivity analyses were added to support this reframing.

We also directly applied the finalized frozen 10-gene classifier, without retraining or outcome-based tuning, to four public HCC immunotherapy cohorts (GSE140901, GSE215011, GSE279750, and GSE202069). These analyses were heterogeneous and imprecise and did not establish validated response prediction. We therefore removed language implying predictive validation or clinical utility and now present the treatment findings as exploratory, treatment-associated evidence.

To address the concern about circular biological validation and to strengthen the less obvious myeloid-oriented end, we added strict non-overlap bulk and single-cell analyses and expanded complementary localization and cross-modal evidence. The revised GSE149614 analysis uses exact cell-identifier matching and sample-aware pseudobulk scoring. PDC000198 and PDC000199 now provide paired protein and phosphoproteomic support for macrophage, fibroblast/ECM, hypoxia, SPP1-integrin-FAK, and myeloid-receptor programs. We explicitly distinguish transcriptomic support derived from strict non-overlap gene sets from characterization that reuses state-defining genes, and we describe cross-modal molecular concordance separately.

The ethical provenance of the GSE245908 human spatial tissue sections is now stated explicitly. The source publication reports that patients were informed about the secondary research use of their data and tissue samples for research purposes and gave consent, and that the study was approved by the Health and Research Ethics Center of Bordeaux (CER-BDX 2024-10). The revised manuscript also clarifies that our work was a secondary analysis of publicly available data and involved no new participant recruitment, tissue collection, or access to identifiable private information.

The Funding Statement has been amended to include the journal-requested sentence, “There was no additional external funding received for this study,” and the same complete statement is included in the revised cover letter. For code availability, a reviewer-access Zenodo record containing the reproducibility package has already been created and the access information is supplied separately with this revision. The record will be released publicly without access restrictions upon publication, at which time the public DOI or permanent link will be inserted in the submission system and final manuscript.

We have also reorganized the manuscript so that methodological detail is concentrated in the Materials and Methods, whereas the Results emphasize findings, effect sizes, uncertainty, and interpretation. We appreciate the Editor's guidance, which has helped us make the manuscript more transparent, reproducible, and appropriately cautious.

Sincerely,

Xiao Chen and Sha Huang

on behalf of all authors

Responses to the Reviewers

Reviewer 1

Major Issues

Comment 1. Biological rationale for the two-state hypothesis is insufficient. The authors assumed that immune-active HCC comprises two discrete immune states but the rationale for this hypothesis is not clearly established. If this model is based on previous literature, the relevant studies should be discussed.

Response: We thank the reviewer for this important comment. We agree that the biological origin of our working hypothesis was not explained sufficiently in the original manuscript and, more importantly, that our previous wording went beyond what the prior literature and our discovery data could establish by describing the two tendencies as discrete immune states.

Our original framework was hypothesis-driven rather than derived solely from the separation observed in TCGA-LIHC. Previous HCC studies suggested that immune-active tumors can contain qualitatively different immune ecologies. Sia et al. identified an immune-specific HCC class that could itself be subdivided into an adaptive T-cell-response subtype and an exhausted subtype characterized by TGFβ1-regulated immunosuppressive programs. Subsequent large-scale single-cell analyses of liver cancer identified distinct tumor immune microenvironment ecologies that included immune activation as well as myeloid- and stromal-mediated immune suppression. Together with studies demonstrating extensive lymphoid, myeloid, stromal, and malignant-cell heterogeneity in HCC, these observations motivated our original two-pole working model: one end characterized by IFNγ-linked lymphoid effector competence and the other by countervailing myeloid/stromal suppressive remodeling.

We agree, however, that this literature supports opposing biological tendencies rather than proving that they must form two naturally discrete patient populations. Prompted by the reviewer’s concern, we therefore directly tested the discreteness assumption rather than retaining it as an untested premise. The newly added mixture-model, kernel-density, and bootstrap analyses showed structured heterogeneity but did not demonstrate robust natural bimodality. We consequently revised the conceptual model throughout the manuscript: the continuous IFNγ-responsive-to-myeloid-suppressive axis is now the primary biological representation, whereas “IFNγ-responsive-associated” and “myeloid-suppressive-associated” are retained only as operational labels for opposing ends of this continuum.

Thus, the revised manuscript preserves the literature-supported biological rationale for the two opposing immune tendencies while no longer claiming that immune-active HCC comprises two inherently discrete immune populations. We have expanded the Introduction to explain the literature basis explicitly and revised the Abstract, Methods, Results, Discussion, Conclusions, title, and figure legends to maintain this distinction consistently.

Location in manuscript: Title (lines 1–2); Introduction—literature basis and continuous-axis framework (lines 60–94); Materials and Methods—Immune-axis definition, operational labels, and sensitivity analyses (lines 167–221); Results—Immune-active HCC is organized along a structured IFNγ-to-myeloid immune continuum (lines 461–514); Discussion—continuum interpretation (lines 738–749) and limitations/claim boundary (lines 815–848); Conclusions (lines 849–862).

Comment 2. The authors defined immune-active tumors using the top 30% of checkpoint expression. The rationale for selecting checkpoint expression as the entry criterion over other established measures (e.g., immune infiltration, cytolytic activity, T-cell activation, immune scores) is unclear.

Furthermore, the data appear to support a continuous immune axis rather than two clearly separated biological populations, as acknowledged by the authors. Given that most tumors cluster within a similar region (Fig. 2B, most samples fell in the lower left region) of the inflamed-versus-myeloid space, the biological justification for imposing a binary classification is not convincing.

Response: We thank the reviewer for raising two closely related concerns: the choice of checkpoint expression as the original immune-active entry criterion and the biological justification for a binary classification. We agree that neither point was sufficiently explained in the original manuscript, and we have revised both the analytical framework and its presentation accordingly.

Checkpoint expression was originally used as a pragmatic enrichment signal for an immune-engaged and immunotherapy-relevant tumor context, rather than as a gold-standard or uniquely preferred definition of immune activity. The historical discovery workflow explored checkpoint-based entry windows spanning the top 20%, 25%, 30%, and 35% of the checkpoint distribution, and the checkpoint-top-30% plus axis-median route was subsequently used to construct the frozen compact classifier. We recognize, however, that the original exploratory route-screening procedure included TCGA survival information and therefore does not establish the top-30% checkpoint threshold as a uniquely optimal biological cutoff. We have made this provenance explicit in the revised manuscript and no longer use TCGA survival performance to justify or validate the immune-active definition.

To test whether the biological findings depended on the exact historical checkpoint cutoff without using outcome information, we added a new checkpoint-threshold sensitivity analysis across the top 20%, 25%, 30%, and 35% windows. These thresholds selected 75, 93, 112, and 130 tumors, respectively. Relative to the historical checkpoint-top-30% subset, the corresponding sample-set Jaccard indices were 0.670, 0.830, 1.000, and 0.862, while operational-label concordance among overlapping samples was 93.3%, 93.5%, 100%, and 97.3%. Importantly, the principal biological polarity was stable across all four thresholds: Hedges' g remained positive for inflamed (1.01-1.27), antigen-presentation (0.76-0.95), and T-cell-support (0.93-1.19) programs and negative for the myeloid program (-0.65 to -0.58). These analyses indicate that the biological polarity is not contingent on the exact 30% cutoff (Fig 2C; Fig S1A,D).

We further evaluated whether the conclusions depended on checkpoint expression as the immune-active entry measure itself. Seven outcome-independent alternatives were tested: an immune composite, the inflamed module, cytolytic activity, T-cell support, CD8 expression, immune infiltration, and a T-cell-inflamed gene-expression profile. Although sample overlap with the historical checkpoint-top-30% subset was only partial (Jaccard 0.534-0.647), the same IFNγ-versus-myeloid biological polarity was preserved across all definitions. We therefore now describe checkpoint-top-30% as the historical discovery entry rule rather than as a unique biological definition of immune-active HCC (Fig 2D; Fig S1B).

We also agree with the reviewer that the discovery data do not establish two naturally separated patient populations. We therefore evaluated the continuous axis using one-, two-, and three-component Gaussian mixtures together with kernel-density and bootstrap stability diagnostics. Among the candidate mixtures, BIC favored the three-component model rather than uniquely supporting a two-component partition; we do not interpret this fitted component count as a corresponding number of biological classes. The observed kernel-density curve contained only one prominent mode, and none of 1,000 nonparametric bootstrap resamples generated with a fixed seed showed two or more prominent density modes. The median bootstrap adjusted Rand index for the two-component assignment was 0.504, indicating moderate and incomplete assignment stability and further supporting our decision to treat the continuous axis as the primary representation. We therefore interpret the mixture fits as evidence of structured non-Gaussian heterogeneity rather than natural class structure, and the continuous IFNγ-responsive-to-myeloid-suppressive axis remains the primary representation.

Accordingly, the continuous inflamed-minus-myeloid axis is now the primary biological representation throughout the revised manuscript. The median split is retained only for transparent group-based descriptive comparisons and compatibility with the already frozen compact-classifier framework; it is not interpreted as evidence of two intrinsically discrete biological populations. In external deployment, samples are also not forced into two categories because the frozen class-specific uncertainty thresholds allow an intermediate group. To preserve the core discovery content of the original manuscript while directly addressing the reviewer's concern, the redesigned Fig 2 now integrates: the continuous TCGA state-space geometry (Fig 2A), continuity/modality diagnostics (Fig 2B), checkpoint-threshold sensitivity (Fig 2C), alternative immune-active entry definitions (Fig 2D), and strict non-overlap biological support after exclusion of all compact-panel and state-defining genes (Fig 2E). Detailed robustness analyses are provided in Fig S1A-D.

Location in manuscript: Abstract (lines 20–46, especially lines 25–31); Materials and Methods—Immune-axis definition, operational labels, and sensitivity analyses (lines 167–221) and Reproducibility safeguards and claim boundaries (lines 435–459); Results—continuity, checkpoint-threshold sensitivity, alternative entry definitions, and strict non-overlap analyses (lines 461–514); Fig 2 legend (lines 476–487); Discussion—continuum interpretation (lines 738–749) and limitations (lines 815–848); Conclusions (lines 849–862). Supporting Information: Fig S1A–D.

Comment 3. The discovery framework is partially circular. The proposed immune states were defined using inflamed and myeloid immune programs, yet subsequent validations showed that IFNγ-responsive state exhibited higher IFNγ signaling, checkpoint activity, antigen presentation, and T-cell-support signatures, while the other showed stronger myeloid programs. These downstream analyses are confirmatory rather than independent validation. Greater emphasis should instead be placed on orthogonal evidence to demonstrate that the proposed states capture novel biology beyond the discovery framework.

Response: We thank the reviewer for identifying this important concern. We agree that analyses reusing the original state-defining biology should be regarded as biological characterization rather than independent validation. We therefore reorganized the evidence hierarchy and added strict non-overlap transcriptomic analyses after excluding all 10 compact-panel genes and all genes in the original checkpoint, inflamed, and myeloid definitions.

In TCGA-LIHC, strict non-overlap antigen-processing and T-cell-effector programs remained higher toward the IFNγ-associated end (Hedges’ g = 0.688 and 0.657; FDR = 3.84 × 10^-4 and 2.93 × 10^-4, respectively). In the reanalyzed GSE149614 dataset, using exact cell-identifier matching and sample-aware pseudobulk analysis, the strict non-overlap antigen-processing program correlated with the frozen 10-gene template-contrast score across 13 tumor-related anatomical samples (Spearman rho = 0.758; raw P = 0.00267; FDR = 0.0133 across five prespecified programs).

The non-overlap transcriptomic evidence was weaker for the myeloid-oriented end, so we do not use transcriptomic concordance alone to establish that ecology. Instead, PDC000198 showed higher strict non-overlap macrophage, fibroblast/ECM, and hypoxia protein programs toward the myeloid-oriented end (FDR = 0.0289, 0.0097, and 0.0025), while PDC000199 showed concordant myeloid-oriented phosphorylation associations for SPP1-integrin-FAK, fibroblast/ECM, myeloid-receptor, and ECM-FAK-SRC programs (FDR = 5.89 × 10^-4, 0.0028, 0.0056, and 0.0464). Analyses that reuse defining genes are now labeled as characterization or localization, whereas strict non-overlap transcriptomic evidence and cross-modal proteomic/phosphoproteomic evidence are described separately.

Location in manuscript: Materials and Methods—Single-cell, spatial, proteomic, and phosphoproteomic analyses (lines 270–347) and Reproducibility safeguards and claim boundaries (lines 435–459); Results—alternative-entry/strict non-overlap bulk analyses (lines 500–514) and single-cell, spatial, proteomic, and phosphoproteomic evidence (lines 559–639); Fig 2E legend (lines 485–487); Fig 4C legend (lines 586–587) and Fig 4E,F legend (lines 591–596); Discussion—biological interpretation and evidence hierarchy (lines 750–796) and cross-modal limitations (lines 827–848); Conclusions (lines 853–862). Supporting Information: Table S1 and S3 and corresponding S1/S3 figures.

Comment 4. The biological and translational significance of the proposed classification remains unclear. While subdividing immune-active HCC is potentially valuable, the manuscript does not demonstrate how this classification advances current understanding or improves clinical decision-making. It remains unclear whether these immune states better predict immunotherapy response, identify distinct therapeutic vulnerabilities, or provide information beyond existing immune classification systems. The multi-omic analyses are largely descriptive and do not substantially extend the biological findings from the discovery cohort. Applying the finalized 10-gene classifier directly to HCC immunotherapy cohorts and evaluating its predictive performance would substantially strengthen the translational impact.

Response: We thank the reviewer for this important point. We agree that the original GSE206325 projection addressed transcriptional treatment relevance rather than the clinically more important question of how the finalized score performs in treated HCC patients. We therefore applied the previously frozen 10-gene template-contrast score, without retraining or outcome-based retuning, to four public HCC immunotherapy cohorts: GSE140901 (n = 24), GSE215011 (n = 10), GSE279750 (n = 10), and GSE202069 (n = 17). Cohort-specific AUCs were 0.510, 0.520, 0.750, and 0.319, and Hedges’ g values were 0.100, 0.151, 0.896, and −0.342, respectively. Random-effects pooling yielded Hedges’ g = 0.10 (95% CI −0.42 to 0.61) and an OR per SD of 1.08 (95% CI 0.62 to 1.89), indicating heterogeneous and imprecise response associations.

Exploratory benchmarking also did not demonstrate superiority of the 10-gene score over simpler immune measures; CXCL9 alone showed a larger pooled positive association (Hedges’ g = 0.59, 95% CI 0.06–1.12). GSE140901 lacked B2M, so we did not impute or retune the model; TCGA B2M-dropout sensitivity showed Pearson r = 0.994 and 92.5% sign concordance.

Accordingly, we removed claims of validated response prediction, incremental predictive value, clinical decision-making benefit, or superiority over established immune signatures. Because the available treated HCC cohorts lack matched non-ICI comparator arms, the results demonstrate response association within treated cohorts rather than treatment-specific predictive value. The multi-omic myeloid/stromal findings are likewise presented as hypotheses for future functional and combination-treatment studies rather than validated therapeutic vulnerabilities.

Importantly, the direct treatment-cohort analysis provides an empirical constraint on the translational interpretation. The locked score was challenged in independent treated HCC cohorts without outcome-based retuning, and the heterogeneous overall null result prevents biological transferability from being converted into an unsupported clinical claim. We therefore define the present translational output as a reproducible research stratification coordinate and a set of testable myeloid/stromal hypotheses, while any treatment-selection utility remains to be established in adequately powered prospective HCC studies.

Location in manuscript: Abstract—treatment-cohort conclusion (lines 41–46); Materials and Methods—Direct HCC immunotherapy-cohort evaluation and meta-analysis (lines 374–420; GSE202069 Cox specification at lines 388–395); Results—Direct HCC treatment-cohort evaluation does not establish clinical response prediction (lines 640–699); Fig 5 legend (lines 651–661); Discussion—treatment-claim boundary and clinical limitations (lines 797–848); Conclusions (lines 857–862). Supporting Information: Fig S4A–F and Table S4.

Comment 5. The classifier largely recapitulates existing immune-active signatures. Several genes included in the 10-gene classifier (IFNG, CXCL9, PRF1) are already widely used to define inflamed or immune-active tumors. Consequently, identifying an IFNγ-responsive subgroup is expected and provides limited additional biological insight. The manuscript would be strengthened by focusing more deeply on the biological characteristics and therapeutic relevance of the myeloid-suppressive subgroup, which represents the more novel aspect of the study.

Response: We thank the reviewer for this important observation. We agree that IFNG, CXCL9, and PRF1 are established markers of an inflamed immune phenotype and that recovery of an IFNγ-oriented end is therefore biologically expected. In the original manuscript, these genes were deliberately retained because the compact panel was intended to preserve an interpretable inflamed anchor while simultaneously encoding opposing myeloid counter-pressure during cross-platform transfer, rather than because we considered the individual IFNγ-related genes to represent novel biomarkers. We agree that our original emphasis on the classifier could obscure this distinction, and we have revised the manuscript accordingly.

The revised manuscript now explicitly describes the 10-gene panel as a compact deployment layer for the biological axis rather than as a uniquely optimal or biologically novel immune signature. We also benchmarked the frozen 10-gene template-contrast score against simpler immune scores, including IFNG, CXCL9, cytolytic activity, checkpoint, inflamed, myeloid-inverse, and inflamed-minus-myeloid scores across the same four HCC immunotherapy cohorts. This analysis did not demonstrate superiority of the frozen 10-gene template-contrast score. CXCL9 alone showed the strongest pooled positive association (Hedges’ g = 0.59, 95% CI 0.06–1.12), whereas the frozen 10-gene template-contrast score showed a smaller and imprecise pooled association (g = 0.10, 95% CI −0.42 to 0.61). We therefore do not claim that the compact classifier improves on established immune signatures for clinical response prediction.

In response to the reviewer’s suggestion, we substantially expanded the biological characterization of the less obvious myeloid-oriented end. Importantly, the transcriptomic evidence from strict non-overlap gene sets was asymmetric: strict non-overlap antigen-processing and T-cell-effector programs predominantly supported the IFNγ-oriented end, whereas strict non-overlap macrophage and fibroblast/ECM transcriptomic programs were weaker or nonsignificant. We therefore do not use transcriptomic concordance alone to establish the myeloid-oriented ecology. Instead, the revised manuscript emphasizes cross-modal evidence. In PDC000198, strict non-overlap macrophage, fibroblast/ECM, and hypoxia protein programs were significantly higher toward the myeloid-oriented end (FDR = 0.0289, 0.0097, and 0.0025, respectively). In PDC000199, SPP1-integrin-FAK, fibroblast/ECM, myeloid-receptor, and ECM-FAK-SRC phosphorylation programs were inversely associated with the separately measured PDC000198 partial protein axis (FDR = 5.89×10⁻⁴, 0.0028, 0.0056, and 0.0464, respectively), with extreme-group analyses providing concordant support for SPP1-integrin-FAK, myeloid-receptor, fibroblast/ECM, and hypoxia-associated signaling. These data shift the biological emphasis of the revised study toward a myeloid-stromal signaling ecology rather than toward rediscovery of an established IFNγ-inflamed phenotype.

We also agree that the therapeutic relevance of this myeloid-oriented biology should be distinguished from demonstrated therapeutic vulnerability. The recurrent macrophage, fibroblast/ECM, hypoxia, SPP1-integrin-FAK, and myeloid-receptor programs generate candidate hypotheses for future combination-treatment studies, but the public datasets analyzed here do not provide perturbational, pharmacologic, or randomized evidence sufficient to establish targetability. We therefore present these pathways as priorities for functional investigation rather than as validated therapeutic targets.

Accordingly, the revised manuscript no longer presents identification of the IFNγ-responsive arm or the individual IFNγ-related classifier genes as the principal novelty. The contribution is instead positioned as a transferable framework for a structured IFNγ-to-myeloid immune continuum, together with deeper multi-layer characterization of the myeloid/stromal end and explicit definition of the current clinical and therapeutic claim boundaries.

Location in manuscript: Results—compact transfer panel and external transfer (lines 515–558) and single-cell/cross-modal evidence (lines 559–639); Fig 4E,F legend (lines 591–596); Fig 5E legend (lines 659–661); Discussion—myeloid/stromal biological interpretation and compact-panel claim boundary (lines 750–796); Conclusions (lines 853–862). Supporting Information: Fig S4E and Table S3 and S4.

Comment 6. The manuscript states that projection into untreated HCC cohorts demonstrates higher responder-up signals and favorable responder-axis shifts in the IFNγ-responsive state. Can the author explain more in the manuscript as to how they arrived at this conclusion?

Further, the distinction between transcriptional similarity to responder programs and validated prediction of immunotherapy benefit should be clarified throughout the manuscript.

Response: We thank the reviewer for asking us to make this derivation explicit. We agree that the original manuscript moved too quickly from the untreated-cohort projection to its interpretation and that the wording could be read as implying treatment-response prediction. The original purpose of this analysis was narrower: it was designed as an HCC-specific biological bridge between the pre-existing immune axis and transcriptional programs associated with anti-PD-1 response in an independent treated HCC dataset, not as a surrogate clinical-validation analysis. We have now made both the derivation and this claim boundary explicit in the Methods, Results, Fig 5 legend, and Supplementary Methods.

Specifically, in GSE206325 we linked source-study response annotations to biological sample identifiers and aggregated cells within each sample to sample-level pseudobulk counts, followed by library-size normalization to counts per million and log1p transformation. The derivation set contained 92 responder-labeled and 176 non-responder-labeled pseudobulk profiles; these counts refer to pseudobulk profiles rather than independent patients. For each gene, we calculated the responder-group mean minus the non-responder-group mean. The 50 genes with the largest positive effects were frozen as the responder-up program and the 50 genes with the largest negative effects as the responder-down program. We then projected these fixed programs into untreated TCGA-LIHC and GSE14520 using within-sample percentile-rank scoring and defined the responder axis as responder-up score minus responder-down score. Using the already assigned operational end-group labels, the IFNγ-associated group showed higher mean responder-up and responder-axis scores than the myeloid-associated group in both untreated cohorts. This descriptive direction was the basis of the original statement.

We now explicitly state that this projection measures transcriptional resemblance to GSE206325 response-associated programs only. TCGA-LIHC and GSE14520 do not provide corresponding immunotherapy-response outcomes for this analysis, so the projection cannot estimate response probability, discrimination, calibration, or clinical benefit. We therefore no longer describe the projected shift as evidence of favorable clinical response. Direct frozen application of the finalized classifier to GSE140901, GSE215011, GSE279750, and GSE202069 is now the primary treatment-related test; those results were heterogeneous and imprecise (four-HCC pooled Hedges’ g = 0.10, 95% CI −0.42 to 0.61) and do not establish validated response prediction. Cross-cancer treatment cohorts remain secondary context only.

Location in manuscript: Materials and Methods—Treatment-program derivation and untreated-HCC projection (lines 348–373) and Direct HCC immunotherapy-cohort evaluation and meta-analysis (lines 374–420); Results—treatment-program bridge and direct HCC treatment-cohort evaluation (lines 640–699); Fig 5A,B legend (lines 652–657); Discussion—treatment-program and clinical claim boundary (lines 797–814). Supporting Information: Supplementary Methods S8 and Table S4G.

Minor Issues

Comment 1. The manuscript states that a biologically curated 10-gene panel was selected but does not adequately explain why these specific genes or why exactly ten genes were chosen. I could not fully extrapolate the information regarding feature selection, robustness, or sensitivity analyses from this manuscript.

Response: We thank the reviewer for highlighting that the original manuscript did not clearly separate candidate-gene curation, panel-size selection, final deployment-rule construction, and sensitivity analyses. We have revised the Methods, Results, Fig 3 description, and Supplementary Methods to make this workflow explicit.

The candidate pool was biologically restricted to genes from the prespecified checkpoint, inflamed, myeloid, antigen-presentation, and T-cell-support modules and was evaluated using the previously assigned operational TCGA end-group labels; treatment outcomes were not used to rank candidate genes or to select panel size. Candidate genes were ranked by the absolute mean standardized-expression difference between the two operational end groups while retaining representation from both biological directions. We then screened candidate panel sizes from 10 through 16 genes by fivefold stratified cross-validation using a nearest-centroid classifier. The historical optimization score was balanced accuracy minus 0.10 times the absolute difference between the two class-specific recalls, which penalized solutions that recovered one end group substantially better than the other. Within this prespecified size range, the 10-gene candidate had the highest internal optimization score and balanced accuracy, whereas adding genes did not improve the screened internal criterion (Fig 3A). We therefore retained five genes from the historical IFNγ-associated selection direction (CXCL9, B2M, IFNG, CTLA4, and PRF1) and five genes from the historical myeloid-associated selection direction (VEGFA, TREM2, TGFB1, APOE, and SPP1). As clarified in the revised manuscript, these historical selection directions refer to the standardized-expression screen used for panel construction; the final frozen template-contrast weights were recalculated after stability-enriched rank recentering and therefore need not preserve the same sign for every individual gene. We now explicitly state that this was a limited panel-size screen and does not establish ten genes, or this exact feature combination, as globally or uniquely optimal. Because panels smaller than 10 genes were not evaluated in the historical screen, we also do not interpret this analysis as establishing 10 genes as the minimum sufficient panel size. Rather, it shows only that the 10-gene candidate performed best within the prespecified 10–16-gene range that was actually tested.

After the panel had been fixed, the cross-platform deployment workflow used within-sample ranks and frozen template-contrast weights with class-specific uncertainty thresholds; external cohorts were not used to reselect genes, change score direction, optimize thresholds, or choose the method. We also clarified the relevant sensitivity analyses. Fig 3A shows the panel-size sensitivity screen, frozen external transfer was evaluated in GSE14520 and ICGC-LIRI-JP, and the missing-gene pattern encountered in GSE140901 was tested by omitting B2M in TCGA. The complete versus B2M-omitted scores were highly concordant (Pearson r = 0.994; Spearman rho = 0.989), with 92.5% sign concordance and 84.1% three-class concordance. These analyses support robustness and portability of the compact deployment layer while also showing why we describe it as a pragmatic transfer device rather than a uniquely optimal or clinically validated predictor. Importantly, feature ranking in the historical development workflow was performed once in the full TCGA discovery subset before the fivefold panel-size screen. We now state this explicitly and treat the cross-validation metric only as a historical engineering heuristic for comparing candidate panel sizes, not as an unbiased estimate of out-of-sample performance. This distinction does not affect the frozen external deployment analyses, in which the finalized panel, direction, templates, and thresholds were not reselected using external outcomes.

Location in manuscript: Materials and Methods—Compact transfer-panel development and external transfer (lines 222–269); Results—A locked 10-gene template-contrast rule provides a portable continuous proxy for axis position across HCC cohorts (lines 515–558); Fig 3A,B legend (lines 529–533). Supporting Information: Supplementary Methods S4, Table S2D, and Table S4D.

Comment 2. Throughout the Results section, conclusions are often difficult to associate with the corresponding figures. More precise figure citations would improve readability.

Response: We thank the reviewer for this helpful suggestion. We systematically audited the Results-to-figure links and revised the text so that quantitative findings and their interpretation are accompanied by the specific figure panel or supplementary-table subsection that supports them, rather than by broad figure-level citations at the end of a paragraph. In particular, continuity and modality are linked to Fig 2A,B; checkpoint-threshold sensitivity to Fig 2C; alternative immune-active definitions and strict non-overlap bulk analyses to Fig 2D,E; panel-size screening and the frozen 10-gene panel to Fig 3A,B; external deployment and biological polarity to Fig 3C,D; single-cell compartment localization, paired tumor shift, and strict non-overlap single-cell analyses to Fig 4A-C; spatial proximity to Fig 4D; proteomic and phosphoproteomic analyses to Fig 4E,F; GSE206325 derivation and untreated-HCC projection to Fig 5A,B; direct HCC response evaluation to Fig 5C,D; and simple-signature benchmarking to Fig 5E. The exploratory GSE202069 survival associations are reported only in Supplementary Table S4B and are not used to support prognostic or predictive claims. Upstream molecular anchoring is linked to Fig 6A-D. We similarly replaced generic Supplementary Table references with subsection-level citations where appropriate. These revisions allow each main conclusion to be traced directly to its supporting analysis.

Location in manuscript: Results—continuity/entry-sensitivity analysis with Fig 2 citations (lines 461–514); compact-panel transfer with Fig 3 citations (lines 515–558); single-cell, spatial, proteomic, and phosphoproteomic evidence with Fig 4 citations (lines 559–639); direct HCC treatment evaluation with Fig 5 citations (lines 640–699); upstream multi-omic anchoring with Fig 6 citations (lines 700–736). Supporting Information: Table S1–S5 and the corresponding Supplementary Figure legends.

Comment 3. Figure 4C alone does not adequately support the conclusions. Supporting analyses currently presented in the Supplementary Figures should be incorporated into the main figures, and regions demonstrating co-localization of IFNγ-like and myeloid-like states should be more clearly annotated.

Response: We thank the reviewer for this helpful suggestion. We agree that the original representative spatial panel alone was insufficient to support the spatial conclusion. We therefore moved the key spatial evidence into the main figure and revised Fig 4D to show both analyzable GSE245908 sections together with a section-wide observed-versus-random-label quantitative contact analysis. Across CHC20 and CHC23, the ecology/reference correlations were 0.196 and 0.259; 75.6% and 83.9% of IFNγ-high spots had a myeloid-high spot within 222 pixels, and the median nearest IFNγ-high-to-myeloid-high distance was approximately 220 pixels in both sections. We additionally made the provenance of the 222-pixel radius explicit. This threshold was not selected from the observed IFNγ-high/myeloid-high distance distribution; it was defined from the native Visium array geometry as the ceiling of the largest direct spot-center lattice-neighbor distance across the two analyzed sections (221.777 pixels). Thus, the primary proximity analysis uses a platform-derived, spot-label-independent neighborhood radius rather than an outcome-tuned spatial cutoff.

Because a high absolute contact fraction can arise simply from endpoint abundance, we further added a count-preserving within-section permutation analysis and now display the observed-versus-null comparison directly in the revised Fig 4D quantitative panel. Under 10,000 random reassignments of the frozen IFNγ-high, myeloid-high, and intermediate labels over the fixed spot coordinates, the expected contact fractions were 90.1% (95% permutation interval, 87.7–92.4%) in CHC20 and 89.9% (87.3–92.5%) in CHC23, whereas the observed fractions were 75.6% and 83.9%, respectively (two-sided empirical P = 2.0 × 10−4 for both sections). We therefore revised the interpretation: opposing programs frequently occur within one Visium-neighbor distance in absolute terms, but the data do not support preferential co-localization; immediate cross-end mixing is lower than expected under an abundance-matched random-label null.

To address the reviewer’s request for clearer spatial annotation while avoiding an overstatement of cellular co-localization, the revised maps now mark representative proximal mixed neighborhoods selected algorithmically from the same neighborhood analysis, i.e., regions in which IFNγ-high spots have nearby myeloid-high spots within the analysis radius. These annotations are illustrative; the inference is based on the full-section quantitative metrics rather than on visually selected regions. Because Visium spots are multicellular and the quantitative results demonstrate neighborhood-level proximity rather than one-to-one cellular overlap, we describe the pattern as local proximity/coexistence rather than definitive cellular co-localization. The more detailed IFNγ, myeloid, and inflamed-minus-myeloid spatial-ecology maps remain in Fig S3C,D.

Location in manuscript: Materials and Methods—spatial analysis, geometry-derived neighborhood radius, permutation null, and algorithmic region annotation (lines 298–334 within the single-cell/spatial methods section); Results—spatial localization and neighborhood analysis (lines 609–623, within lines 559–639); Fig 4D legend (lines 587–591). Supporting Information: Fig S3C,D and Table S3D.

Comment 4. Manuscript lacks overarching roadmap. It would be helpful to sufficiently map out the logic of the paper when describing Fig.1 to orientate the readers.

Response: We thank the reviewer for this helpful suggestion. We agree that the original Introduction and Fig 1 presented the major analytical components but did not make their sequence, analytical roles, and evidence hierarchy sufficiently explicit. We therefore rewrote the final paragraph of the Introduction and redesigned Fig 1 as an explicit five-stage roadmap: (i) discovery of the immune axis, continuity/modality assessment, entry-definition sensitivity analyses, and strict non-overlap bulk characterization; (ii) compact classifier construction and frozen external HCC transfer; (iii) complementary sample-aware single-cell, spatial, proteomic, and phosphoproteomic characterization; (iv) treatment-relevance analysis, separating the GSE206325 responder-program bridge from direct frozen evaluation in four HCC immunotherapy cohorts, with cross-cancer analyses retained only as secondary context; and (v) upstream multi-omic anchoring. The revised Fig 1 also identifies the primary output and claim boundary of each stage and places the continuous IFNγ-responsive-to-myeloid-suppressive axis as the organizing construct. We believe these changes make the analytical flow and the hierarchy of evidence clear at the outset and allow readers to distinguish biological characterization and transferability from exploratory treatment-response analyses.

Location in manuscript: Introduction—overarching analytical roadmap immediately preceding Fig 1 (lines 75–94); Fig 1 legend (lines 95–107).

Reviewer 2

Comment 1. What is the gold standard of this study? The authors appear to have relied solely on assumptions, with no apparent gold standard. There is no wet-lab validation; the findings are derived only from database analysis.

Response: We thank the reviewer for emphasizing the distinction between computational reproducibility, functional validation, and clinical validation. To our knowledge, no externally validated reference standard exists for the specific IFNγ-responsive-to-myeloid-suppressive HCC immune continuum examined here; accordingly, the revised study is not presented as a classification-accuracy study against a gold-standard label.

To strengthen the biological evidence without simply reusing the defining genes, we added outcome-independent entry-definition sensitivity analyses, continuity/modality testing, strict non-overlap transcriptomic analyses, frozen transfer to independent HCC cohorts, sample-aware single-cell and quantitative spatial characterization, and paired proteomic/phosphoproteomic analyses. We also directly evaluated the frozen score in four public HCC immunotherapy cohorts. The pooled treatment association was modest and imprecise (Hedges’ g = 0.10, 95% CI −0.42 to 0.61), and the score did not show clear superiority over simpler immune measures.

We agree that these retrospective public-data analyses cannot substitute for functional perturbation or prospective clinical validation. Functional studies will be required to test causality and therapeutic actionability of the recurrent myeloid/stromal programs, whereas prospective pretreatment HCC cohorts with harmonized treatment and response assessment, ideally including suitable comparator designs, will be required to evaluate treatment-specific predictive utility. We therefore limit the present conclusions to biological organization, cross-dataset transferability, cross-modal support, and hypothesis generation.

Location in manuscript: Materials and Methods—Public datasets and overall study design (lines 110–159) and Reproducibility safeguards and claim boundaries (lines 435–459); Results—strict non-overlap bulk analysis (lines 500–514), single-cell/spatial/cross-modal evidence (lines 559–639), and direct HCC treatment-cohort analysis (lines 640–699); Discussion—biological evidence hierarchy, treatment-claim boundary, and limitations (lines 750–848); Conclusions (lines 849–862).

Comment 2. Some of the content currently in the Results section should be placed in the Methods section. A substantial portion of the Results is written in a style more appropriate for the Methods. The authors appear to have an unclear understanding of the distinction between Methods and Results.

Response: We thank the reviewer for this important comment and agree that the original Results contained excessive procedural detail. We therefore reorganized the manuscript so that analytical definitions, preprocessing, model construction, continuity/modality testing, single-cell and spatial procedures, treatment-program derivation, direct HCC treatment-cohort evaluation, meta-analysis, and upstream multi-omic procedures are described in the Materials and Methods and Supplementary Methods.

We then reviewed all Results subsections and removed or shortened procedural descriptions, retaining only the minimum context needed to identify each frozen analysis. The Results now focus on observed findings, effect sizes, confidence intervals, robustness analyses, and interpretation. Numerical modality findings, including BIC comparisons, kernel-density mode results, bootstrap assignment stability, and the 56/56 operational group counts, are reported in the Results rather than duplicated in the Methods.

Location in manuscript: Materials and Methods—Immune-axis definition and sensitivity analyses (lines 167–221); compact transfer-panel development (lines 222–269); single-cell/spatial/proteomic/phosphoproteomic analyses (lines 270–347); treatment-program projection (lines 348–373); direct HCC immunotherapy evaluation/meta-analysis (lines 374–420); upstream multi-omic anchoring and statistical testing (lines 421–434). Results are separated into finding-focused subsections at lines 460–736. Supporting Information: detailed implementation in Supplementary Methods S3–S10.

Comment 3. In Figure 4C, human tissue sections are presented. Although this is only one component of the validation, it still involves liver tissue sections and requires ethical approval. This issue cannot be overlooked!

Response: We thank the reviewer for identifying this important omission. We agree that, although our study reanalyzes publicly available data, this does not remove the need to report explicitly the ethical provenance of the human tissue from which the spatial-transcriptomic data were generated. In the original submission, this source-study ethics information was not stated sufficiently clearly, and we have corrected this in the revised manuscript.

The human tissue-section component referred to by the reviewer is derived from GSE245908, the spatial-transcriptomics subseries of the source HCC study, and is shown in revised Fig 4D. The two Visium tumor sections reanalyzed here are GSM7850822 (CHC20) and GSM7850823 (CHC23). The original investigators reported that HCC and adjacent non-tumoral tissues were obtained from patients undergoing liver resection at Haut-Lévêque Hospital, Pessac, France. The source publication states that patients were informed about the secondary research use of their data and tissue samples and gave consent, and that the study was approved by the Health and Research Ethics Center of Bordeaux (reference CER-BDX 2024-10).

We have now added these details explicitly to the Materials and Methods section. The present study involved only secondary analysis of publicly available data and involved no new participant recruitment, tissue collection, or access to identifiable private information. Accordingly, no additional institutional ethics approval or informed consent was required for the present secondary analysis.

Location in manuscript: Materials and Methods—Public datasets and overall study design, including identification of the two GSE245908 Visium sections and source-study ethics and consent provenance (lines 110–159, especially lines 138–149). The human spatial tissue sections are shown in revised Fig 4D (legend lines 587–591).

Comment 4. The foundation of this study is based on a binary classification approach. However, the subsequent interpretation employs "frozen state-axis scores". This appears to be contradictory.

Response: We thank the reviewer for identifying this important conceptual ambiguity. We agree that the terminology in the original manuscript made a discrete discovery label and a continuous deployment score appear contradictory, and we have revised the manuscript to distinguish these two levels explicitly. In the historical workflow, the median split of the inflamed-minus-myeloid axis provided a pragmatic and balanced set of operational end-group labels for construction and internal evaluation of a compact cross-platform transfer model. That labeling step should not, however, be interpreted as evidence that the underlying biology is intrinsically binary.

We therefore tested this assumption directly in the revision. Within the historical checkpoint-high TCGA subset, BIC favored the three-component Gaussian-mixture model among the one- to three-component candidates, while the two-component model also improved on the one-component model. We do not interpret the fitted mixture-component count as a corresponding number of biological classes. The observed kernel-density curve contained one prominent mode, none of 1,000 nonparametric bootstrap resamples generated with the fixed seed specified in the deposited code showed two or more prominent density modes, and the median bootstrap adjusted Rand index for the two-component assignment was 0.504, indicating moderate and incomplete assignment stability and further supporting our decision to treat the continuous axis as the primary representation. These results indicate structured heterogeneity without robust empirical multimodality and do not support two naturally separated populations. Accordingly, the continuous inflamed-minus-myeloid axis is now the primary biological representation, and the median-split IFNγ-responsive-associated and myeloid-suppressive-associated labels are retained only as operational end-group summaries and historical targets for compact-panel construction.

We also now distinguish the biological discovery axis from the frozen 10-gene template-contrast score used for cross-platform deployment. The discovery axis is the continuous difference between the prespecified inflamed and myeloid module scores. The frozen template-contrast score is a separate continuous, signed 10-gene rank-based output constructed from the operational end-group labels and oriented in the same IFNγ-to-myeloid direction. Fixed class-specific thresholds are applied only when categorical operational labels are required; samples falling between these thresholds remain intermediate. Thus, the continuous transfer score and the thresholded operational labels are not competing biological definitions: the former is the continuous deployment output, whereas the latter are optional discretized summaries of that score. Direct treatment-cohort analyses use the continuous frozen template-contrast score rather than forcing samples into two classes.

We have revised the Abstract, Introduction, Materials and Methods, Results, Fig 3, and Supplementary Methods to make this hierarchy explicit and to avoid implying that the compact classifier establishes two naturally discrete biological populations.

Location in manuscript: Abstract—continuous-axis framing (lines 25–46); Introduction—revised analytical framework (lines 75–94); Materials and Methods—Immune-axis definition, operational labels, and sensitivity analyses (lines 167–221) and Compact transfer-panel development and external transfer (lines 222–269, especially lines 250–260); Results—continuity/modality analysis and compact-panel transfer (lines 461–558); Fig 2A,B legend (lines 477–482) and Fig 3C legend (lines 533–535); Discussion—continuum interpretation (lines 738–749). Supporting Information: Supplementary Methods S3 and S4 and Table S2.

Comment 5. There is almost no clinical validation, and the findings do not fully or conclusively support the authors' conclusions.

Response: We agree with the reviewer that the original manuscript did not contain sufficient clinical evidence to support a validated response-prediction claim. We therefore made two substantive changes: we added direct frozen evaluation in the available public HCC immunotherapy cohorts, and we revised the clinical conclusions to follow the results of those analyses rather than extrapolating beyond them.

The finalized 10-gene template-contrast score was applied without retraining, gene reselection, direction changes, threshold optimization, response redefinition, or other outcome-based tuning to four public HCC immunotherapy cohorts: GSE140901, GSE215011, GSE279750, and GSE202069. The cohort-specific effects were heterogeneous, and the four-HCC pooled association was modest and imprecise (Hedges’ g = 0.10, 95% CI −0.42 to 0.61). Given the small number of available HCC treatment cohorts, we also performed a REML plus Hartung–Knapp small-k sensitivity analysis; the pooled point estimate remained 0.10 while the 95% confidence interval widened to −0.60 to 0.80, reinforcing the same conservative inferential conclusion. Exploratory benchmarking against simpler immune measures also did not demonstrate superiority of the 10-gene score; CXCL9 alone showed a larger pooled positive association. We therefore do not interpret these analyses as validation of a clinically useful response predictor.

We also distinguish association within treated cohorts from treatment-specific prediction. Because the available HCC datasets do not contain appropriate matched non-ICI comparator arms, they cannot establish a biomarker-by-treatment interaction or distinguish treatment-specific predictive value from prognostic or other response-associated biology. Accordingly, we removed language implying validated predictive performance, incremental clinical value, treatment-selection utility, or readiness for clinical implementation. The clinical conclusion of the revised manuscript is deliberately conservative: the currently available public HCC treatment data do not establish a validated clinical response predictor.

Prospective pretreatment HCC cohorts with harmonized treatment and response assessment will be required to determine whether the axis has clinical predictive utility. Such validation should ideally include sufficiently powered cohorts and, where feasible, appropriate non-ICI or alternative-treatment comparator arms to distinguish treatment-specific predictive value from prognostic or other response-associated effects. We now identify this explicitly as a necessary future clinical-validation step before any claim of clinical use, rather than implying that the present retrospective treatment-cohort analyses provide prospective validation.

Location in manuscript: Abstract—treatment-cohort conclusion (lines 41–46); Introduction—analytical framework and claim boundary (lines 87–94); Materials and Methods—Direct HCC immunotherapy-cohort evaluation and meta-analysis (lines 374–420); Results—Direct HCC treatment-cohort evaluation does not establish clinical response prediction (lines 640–699); Fig 5C–E legend (lines 657–661); Discussion—treatment-claim boundary and limitations (lines 797–848); Conclusions (lines 857–862). Supporting Information: Fig S4A–E, Table S4A–S4E and S4H, and Supplementary Methods S8.

Reviewer 3

Comment 1. The current stratification does not sufficiently demonstrate two naturally distinct immune states. Unless functional validation is added, more neutral terms such as the following would be better: IFNγ-responsive-associated state, Myeloid- suppressive-associasted state.

Response: We thank the reviewer for this constructive suggestion and agree that the original terminology implied a degree of natural discreteness that was not established by the data. We therefore adopted the more neutral terms “IFNγ-responsive-associated” and “myeloid-suppressive-associated” and now use these labels only as operational summaries of opposing tendencies along a continuous immune axis.

We also directly tested the discreteness assumption. Although Gaussian-mixture models captured non-Gaussian structure, the observed kernel-density curve contained only one prominent mode, none of 1,000 bootstrap resamples showed two or more prominent modes, and the median bootstrap adjusted Rand index for the two-component assignment was 0.504. These results do not support two naturally separated biological populations. Accordingly, the continuous axis is now the primary representation throughout the manuscript, while thresholded labels are retained only where an operational categorical summary is required. Because no new functional experiments were performed, we do not present the revised terminology or computational analyses as functional validation of discrete immune states.

Location in manuscript: Title (lines 1–2); Abstract (lines 20–46) and Keywords (lines 47–49); Introduction—literature-motivated two-pole hypothesis and continuous-axis framework (lines 60–94); Materials and Methods—terminology and overall analytical framing (lines 110–137, especially lines 124–137) and Immune-axis definition, operational labels, and sensitivity analyses (lines 167–221); Results—continuous-axis analysis and Fig 2 (lines 461–514; Fig 2 legend lines 476–487); Discussion—continuum interpretation (lines 738–749) and study limitations (lines 815–848); Conclusions (lines 849–862). Supporting Information: Supplementary Methods S2 and S3 and Fig S1C.

Additional editorial requirements

1. PLOS ONE style and file-format requirements

Response: We thank the Editor for this reminder. We have rechecked the revised submission against the current PLOS ONE formatting and revision requirements. A clean revised manuscript and a marked-up manuscript with tracked changes are provided, continuous line numbering and page numbering are included, and figure citations and legends have been checked for read-order consistency. Main-figure files will be uploaded using PLOS-compatible figure formats and filenames that match the manuscript labels (Fig1–Fig6), and the Supporting Information files are named consistently with the Supplementary Materials and Supplementary Tables captions in the manuscript.

Location in submission: Clean Manuscript; Revised Article with Changes Highlighted; Fig1–Fig6; Supplementary Materials; Supplementary Tables.

2. Code availability and reproducibility

Response: We thank the Editor for emphasizing unrestricted availability of the author-generated code. A reviewer-access Zenodo record has already been created, and the reviewer-access information is supplied separately with the revision so that the analysis package can be assessed during peer review without exposing the private preview token in the Response to Reviewers. The repository contains the author-generated code and machine-readable provenance required for the principal analyses, including cohort preprocessing and inclusion manifests, immune-active entry sensitivity, continuity/modality analyses, compact-panel construction and frozen deployment, single-cell and spatial analyses, GSE206325 responder-program derivation and untreated-HCC projection, direct HCC treatment-cohort evaluation, comparator benchmarking, proteomic/phosphoproteomic analyses, meta-analysis, figure-generation code, environment/dependency information, frozen model specifications, and provenance records. The public Zenodo record will be released without access restrictions upon publication, and its DOI or permanent link will then be inserted in the submission system and final manuscript.

Location in manuscript: Declarations—Data availability (lines 876–890); Code availability (lines 891–907).

3. Funding disclosure

Response: We thank the Editor for this request. During revision, we rechecked the official award records and identified an error in the recipient initials reported in the original Financial Disclosure. The funding sources and grant numbers are unchanged; only the award-recipient attribution has been corrected. The correct attribution is Natural Science Foundation of Fujian Province grant 2023J011295 to L.L. and grant 2023J011296 to S.H., and Joint Funds for the Innovation of Science and Technology, Fujian Province grant 2021Y9218 to S.H. The complete amended Funding Statement, including the funder website, the funders’ role, and the journal-requested sentence “There was no additional external funding received for this study,” is provided in the revised Cover Letter.

Location in submission: Cover Letter—Funding Statement; online Financial Disclosure updated as requested.

Additional clarification regarding human-participant provenance

Response: In response to the reviewer’s concern regarding the spatial-transcriptomic tissue sections, we also added the source-study ethics and consent information for GSE245908. The manuscript now identifies the two reanalyzed Visium sections, reports the source-study ethics approval (CER-BDX 2024-10) and consent information, and clarifies that the present work was a secondary analysis of publicly available data involving no new participant recruitment, tissue collection, or access to identifiable private information.

Location in manuscript: Materials and Methods—Public datasets and overall study design, especially source-study ethics and consent provenance (lines 138–149).

Attachment Submitted filename: Response to Reviewers.docx

# Peer review: Decision Letter 1

**DOI:** 10.1371/journal.pone.0359327.r003

**Authors:** Kangkang Ji

13 Sep 2026

Integrated multi-omic analysis characterizes an IFNγ-responsive-to-myeloid-suppressive immune continuum in immune-active hepatocellular carcinoma

PONE-D-26-24025R1

Dear Dr. Xiao,

We’re pleased to inform you that your manuscript has been judged scientifically suitable for publication and will be formally accepted for publication once it meets all outstanding technical requirements.

Within one week, you’ll receive an e-mail detailing the required amendments. When these have been addressed, you’ll receive a formal acceptance letter and your manuscript will be scheduled for publication.

An invoice will be generated when your article is formally accepted. Please note, if your institution has a publishing partnership with PLOS and your article meets the relevant criteria, all or part of your publication costs will be covered. Please make sure your user information is up-to-date by logging into Editorial Manager at Editorial Manager® and clicking the ‘Update My Information' link at the top of the page. For questions related to billing, please contact billing support.

If your institution or institutions have a press office, please notify them about your upcoming paper to help maximize its impact. If they’ll be preparing press materials, please inform our press team as soon as possible -- no later than 48 hours after receiving the formal acceptance. Your manuscript will remain under strict press embargo until 2 pm Eastern Time on the date of publication. For more information, please contact onepress@plos.org.

Kind regards,

Kangkang Ji, Ph.D., M.D.

Academic Editor

PLOS One

Additional Editor Comments (optional):

Reviewers' comments:

Reviewer's Responses to Questions

**Comments to the Author**

Reviewer #2: All comments have been addressed

Reviewer #3: All comments have been addressed

**********

2. Is the manuscript technically sound, and do the data support the conclusions?

Reviewer #2: Yes

Reviewer #3: Partly

**********

3. Has the statistical analysis been performed appropriately and rigorously?

Reviewer #2: Yes

Reviewer #3: Yes

**********

4. Have the authors made all data underlying the findings in their manuscript fully available?

The PLOS Data policy

Reviewer #2: Yes

Reviewer #3: Yes

**********

5. Is the manuscript presented in an intelligible fashion and written in standard English?

Reviewer #2: Yes

Reviewer #3: Yes

**********

Reviewer #2: The authors have addressed all of my comments. I have no further comments. My decision is: Accept. Congratulations.

Reviewer #3: (No Response)

**********

what does this mean?). If published, this will include your full peer review and any attached files.

If you choose “no”, your identity will remain anonymous but your review may still be made public.

**Do you want your identity to be public for this peer review?**  For information about this choice, including consent withdrawal, please see our Privacy Policy

Reviewer #2: No

Reviewer #3: No

**********

# Editor report: Acceptance letter

**DOI:** 10.1371/journal.pone.0359327.r004

**Authors:** Kangkang Ji

PONE-D-26-24025R1

PLOS One

Dear Dr. Chen,

I'm pleased to inform you that your manuscript has been deemed suitable for publication in PLOS One. Congratulations! Your manuscript is now being handed over to our production team.

At this stage, our production department will prepare your paper for publication. This includes ensuring the following:

* All references, tables, and figures are properly cited

* All relevant supporting information is included in the manuscript submission,

* There are no issues that prevent the paper from being properly typeset

You will receive further instructions from the production team, including instructions on how to review your proof when it is ready. Please keep in mind that we are working through a large volume of accepted articles, so please give us a few days to review your paper and let you know the next and final steps.

Lastly, if your institution or institutions have a press office, please let them know about your upcoming paper now to help maximize its impact. If they'll be preparing press materials, please inform our press team within the next 48 hours. Your manuscript will remain under strict press embargo until 2 pm Eastern Time on the date of publication. For more information, please contact onepress@plos.org.

You will receive an invoice from PLOS for your publication fee after your manuscript has reached the completed accept phase. If you receive an email requesting payment before acceptance or for any other service, this may be a phishing scheme. Learn how to identify phishing emails and protect your accounts at https://explore.plos.org/phishing.

If we can help with anything else, please email us at customercare@plos.org.

Thank you for submitting your work to PLOS One and supporting open access.

Kind regards,

PLOS One Editorial Office Staff

on behalf of

Dr. Kangkang Ji

Academic Editor

PLOS One
